# Neuromonitoring modalities predicting neurological impairment in pediatric congenital heart disease: a systematic review

**DOI:** 10.3389/fneur.2024.1502762

**Published:** 2024-12-18

**Authors:** Liselotte Van Loo, Bjorn Cools, Anneleen Dereymaeker, Katrien Jansen

**Affiliations:** ^1^Department of Development and Regeneration, KU Leuven, Leuven, Belgium; ^2^Department of Pediatrics, University Hospitals Leuven, Leuven, Belgium; ^3^Department of Cardiovascular Sciences, KU Leuven, Leuven, Belgium; ^4^Department of Pediatrics, Pediatric Cardiology Unit, University Hospitals Leuven, Leuven, Belgium; ^5^Department of Pediatrics, Neonatal Intensive Care Unit, University Hospitals Leuven, Leuven, Belgium; ^6^Department of Pediatrics, Pediatric Neurology Unit, University Hospitals Leuven, Leuven, Belgium

**Keywords:** neuromonitoring, neurodevelopment, neuroimaging, congenital heart disease, near-infrared spectroscopy, electroencephalography, biomarkers

## Abstract

The increased risk of neurodevelopmental impairment in children with congenital heart disease (CHD) has been established, but the search for targeted neurological predictors of adverse outcome is ongoing. This systematic review reports on the utility of three functional neuromonitoring modalities, Near-infrared Spectroscopy (NIRS), electroencephalography (EEG) and biochemical biomarkers, in predicting either clinical neurodevelopmental outcome or structural brain abnormalities after pediatric CHD surgery. Medline, Embase, CENTRAL, Web of Science, clinicaltrials.gov and ICTRP were systematically searched for eligible articles. Original research articles, written in English, published before November 2023 and reporting on perioperative NIRS, EEG or biomarkers and their association with clinical neurodevelopmental outcome or neuroimaging in children <17 years undergoing surgery for CHD were included. The search yielded 11,367 citations, of which 40 papers were included in the final review: sixteen articles (*n* = 908 cases) reported on NIRS, twelve (*n* = 1,163) on EEG and fifteen (*n* = 903) on biochemical biomarkers. Three papers reported on a combination of modalities. Median age at time of surgery was 9 (IQR 7–57) days. Postoperative MRI was performed before discharge at varying timepoints. Median age at clinical outcome assessment was 15 (IQR 12–24) months. Limited evidence supports an association of cerebral oxygen extraction, cerebral desaturation and cerebral autoregulation with outcome, but there was significant heterogeneity in results. Perioperative electroencephalographic ictal discharges and abnormal background were associated with impaired neurological outcome and abnormal neuroimaging. Numerous biochemical biomarkers have been reported but showed no consistent relationship with outcome, except for lactate, which could serve as a predictor of poor outcome. There is a need for larger homogeneous cohorts of children with CHD to determine which perioperative modalities might serve as predictors of neurodevelopmental outcome or neuroimaging abnormalities.

**Systematic review registration:**

http://www.crd.york.ac.uk/PROSPERO, CRD42023479344

## 1 Introduction

Congenital heart disease (CHD) is the most common birth defect with a prevalence of about 8 (1–8) per 1,000 live births (9). Around 25–50% of patients with CHD require neonatal cardiac intervention. Medical and surgical advancements have significantly decreased mortality and morbidity, although, survivors of critical CHD remain at risk of neurodevelopmental impairments in several domains, including overall intellectual functioning, speech, language, executive and memory function, gross, fine motor and visual spatial skills (10). Additionally, around a third of children with congenital heart disease have brain abnormalities on preoperative Magnetic Resonance Imaging (MRI), with an additional third acquiring new or increased injury postoperatively (11). The predominant lesions visualized in postoperative patients with CHD are stroke and white matter injury (1, 12, 13). In addition, children without overt lesions have structural abnormalities such as altered brain volumes and cortical folding on fetal and neonatal brain MRI (2–4). Reviews have shown that perioperative cerebral findings can be associated with neurodevelopmental outcome (NDO) although abnormal neuroimaging is not always proportionally associated with clinical outcome and should be interpreted with caution (5–7). The cause of this neurological risk is considered multi-factorial. Inherent disease- (type of cardiopathy, cyanosis) and patient-specific factors such as genetic syndromes or extracardiac anomalies contribute to the neurological risk profile of these infants (8, 14), in addition to the inherent risk of surgical techniques such as atrial septostomy, cardiopulmonary bypass (CPB) or deep hypothermic circulatory arrest (DHCA) (15, 16), postoperative critical illness and low cardiac output syndrome (17).

Because of this inherent risk, the American Heart Association issued recommendations for the follow-up of neurodevelopment in children with CHD (18). As neurodevelopmental follow-up is a resource-intensive practice, it is most important to identify CHD patients at highest risk for impaired neurodevelopment. Current practices focus largely on clinical development, which is only a late predictor, or on brain MRI which can be performed early but is resource-intensive and not easily accessible in an intensive-care setting. In order to allocate these resources to the patients in greatest need, it is necessary to explore different predictive strategies differentiating subsequent neurodevelopmental risk in CHD patients.

Neuromonitoring practices in a pediatric cardiac intensive-care setting vary widely (19). A recent European survey (20) showed similar variety in perioperative neuromonitoring/neuroimaging after pediatric congenital heart disease surgery: near-infrared spectroscopy (NIRS) was most commonly used, with 64% of centers indicating preoperative, 80% intraoperative and 72% postoperative use. Amplitude-integrated electroencephalography (aEEG) was used in 32% of participating centers, and 20% performed postoperative aEEG. Twelve percent of centers performed preoperative continuous EEG (cEEG), none used it in the postoperative period. Twenty percent of centers measured biochemical biomarkers in the postoperative period. Half of the participating centers indicated having a follow-up program in place for children with CHD.

This systematic review provides a comprehensive overview of the available evidence on three perioperative neuromonitoring modalities, NIRS, EEG and biochemical biomarkers, and their association with subsequent clinical neurological outcome or neuroimaging.

## 2 Methods

### 2.1 Design

This systematic review was conducted in accordance with the Preferred Reporting Items for Systematic Reviews and Meta-Analyses (PRISMA) 2020 guidelines (21). The search strategy was created with help of the biomedical reference librarians of the KU Leuven Libraries – 2Bergen. The protocol was prospectively registered in the international prospective register of systematic reviews (PROSPERO) database (Registration number: CRD42023479344, http://www.crd.york.ac.uk/PROSPERO).

### 2.2 Eligibility criteria

Studies eligible for inclusion reported on the use of neuromonitoring modalities (EEG, NIRS and/or non-invasive biochemical biomarkers) in pediatric patients with CHD necessitating surgical intervention (excluding catheter interventions), and their association with either clinical neurodevelopmental outcome (NDO) evaluated with a validated scale, instrument or test, or postoperative brain MRI evaluating either brain damage or brain maturation. Studies were excluded if the full text was not available in English. Case reports, case series, conference abstracts and review papers were excluded from the analysis.

### 2.3 Search strategy and data sources

We comprehensively searched Medline, Embase, CENTRAL, Web of Science, clinicaltrials.gov and the International Clinical Trials Registry Platform (ICTRP) for eligible studies on November 28th, 2023. The full search strategy can be found in the Supplementary materials. Additionally, we hand-searched references of included studies for relevant publications. References for the selected studies were managed in the Rayyan© software.

### 2.4 Data extraction

After removal of duplicates, all studies were screened based on title and abstract by 2 reviewers (LVL, KJ). Subsequently, the full text of the remaining articles was examined in parallel by 2 reviewers (LVL, KJ) to determine if all inclusion criteria were met. Additionally, the reference list of included articles was manually checked for additional studies. Disagreement was resolved through discussion until consensus was achieved.

One reviewer (LVL) performed data extraction from the manuscripts. The extracted data was summarized in a data extraction sheet. If insufficient data was available from the manuscript, an attempt was made to contact corresponding authors to obtain additional information.

### 2.5 Data analysis

We summarized data on study design, patient characteristics and type of interventions. Primary outcome was either neurodevelopment assessed with standardized neurodevelopmental testing using a validated test, or postoperative neuroimaging using MRI. Papers who did not report standardized assessments (e.g., chart review for neurodevelopmental impairment) were excluded.

For synthesis of the results, studies were grouped by the different neuromonitoring modalities utilized: EEG, NIRS and biochemical biomarkers. It was not possible to perform a meta-analysis due to the large heterogeneity in interventions, reported outcomes and statistical analyses.

### 2.6 Assessment of risk of bias and grading of evidence

Individual studies were assessed for risk of bias using the validated Risk Of Bias In Non-randomized Studies – of Exposure (ROBINS-E) tool for observational data (22). The quality of evidence was assessed for each outcome using the Grading of Recommendations Assessment, Development and Evaluation (GRADE) approach (23), rating the quality of evidence as high, moderate, low or very low in five areas: risk of bias, inconsistency, indirectness, imprecision and publication bias. We aimed to minimize reporting bias by searching clinical trial registries to incorporate unpublished reports.

## 3 Results

### 3.1 Study selection

**Figure 1:**
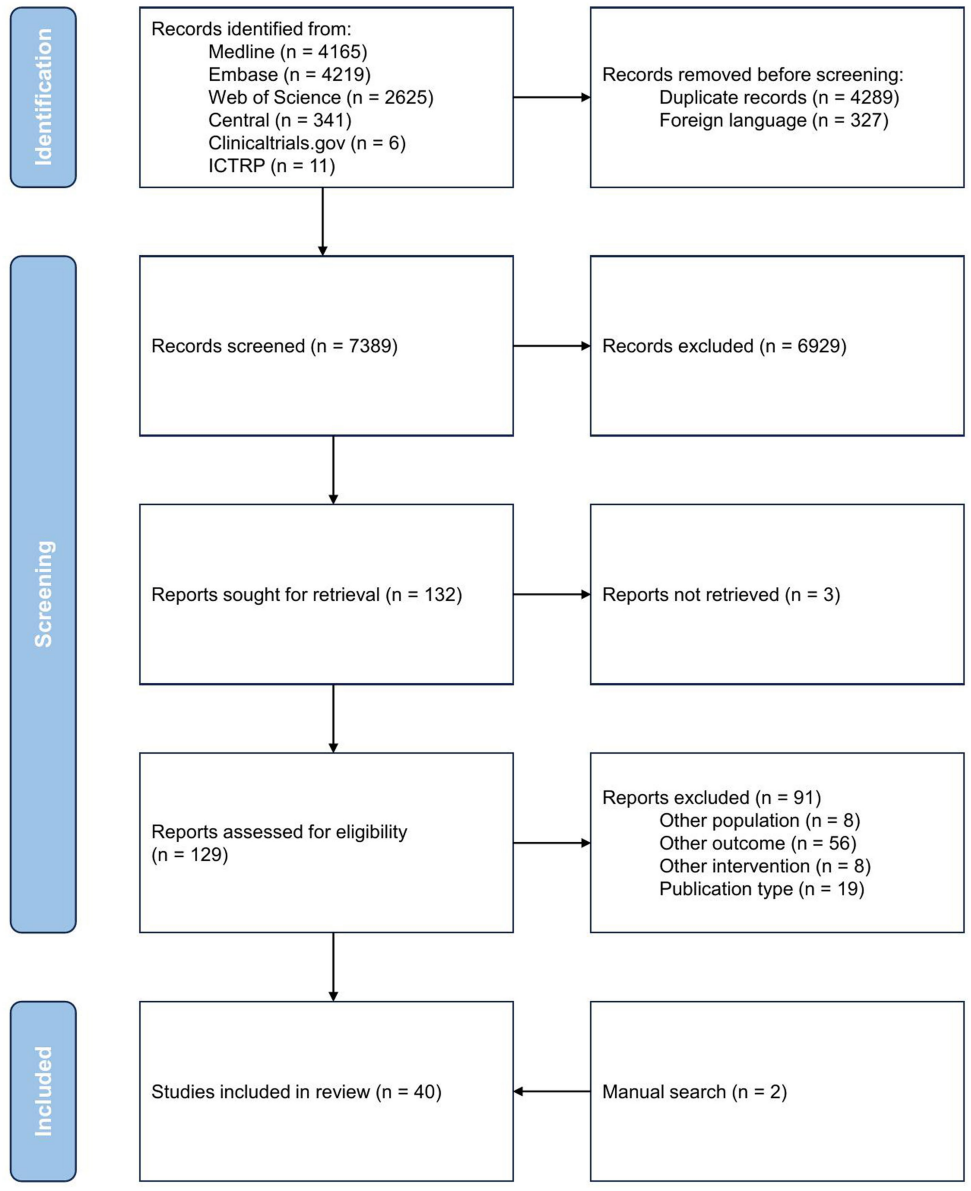
PRISMA flow diagram.

The study selection process is presented in the PRISMA flow diagram (Figure 1). Of the 7,389 records screened, 129 were assessed for eligibility based on full text. We excluded 91 further records: 59 reported on outcomes other than specified in the inclusion criteria (e.g., nonstandardized neurodevelopmental outcome, neuroimaging other than brain MRI), eight articles included different populations (e.g., cardiopathy other than congenital, adults), eight compared interventions than NIRS, EEG or biochemical biomarkers, and 19 articles used non-suitable publication formats (e.g., case reports, case series, review articles). Ultimately, 40 studies met all inclusion criteria and were included in this systematic review (24–63).

Williams et al. (64) performed neonatal high-density, 128-lead, EEG (hdEEG) measurements in children undergoing neonatal cardiac surgery for CHD and measured power (measure of local neural synchrony) and coherence (measure of functional connectivity) as measures of cortical function. While seemingly meeting the inclusion criteria, we excluded this article as the hdEEG is predominantly used in research settings and not deemed feasible in daily clinical practice.

### 3.2 Characteristics of included studies

All studies were observational. Twenty five studies reported prospectively collected data (24–26, 29, 32–35, 37, 41–43, 45–48, 50, 51, 53–56, 58–60, 63). In 14 studies, data was collected retrospectively (27, 28, 30, 31, 36, 38–40, 44, 49, 52, 57, 61, 62).

Most studies had an upper limit for age at inclusion, varying from 30 days to 17 years, median age at surgery was 9 (IQR 7–57) days. 36/40 studies only included patients with critical CHD whereas 4/40 studies included patients with varying disease severity. 25/40 studies specified the necessity for CPB as an inclusion criterium (24, 26–29, 32, 34, 36–39, 41, 43, 45–47, 50, 54, 56, 57, 59–63). In five studies, measurements were performed surrounding a specific procedure (e.g., stage 1 palliation) and five other studies included only specific cardiac diagnoses [mostly hypoplastic left heart syndrome (HLHS) or dextrotransposition of the great arteries (D-TGA)]. All but one study (28) excluded patients with pre-existing neurologic or genetic comorbidities in order to minimize the influence of other causes of neurodevelopmental impairment.

Sixteen studies reported on NIRS as a predictor, with eight studies reporting clinical neurodevelopmental testing (24–31), four studies using MRI as an outcome marker (32–35), and four studies using a combination of both (36–39). aEEG was utilized in five studies (40–44) and seven studies used cEEG (45–51). Of these, outcome variables were clinical neurodevelopment in nine studies (40–43, 45–49), MRI in two (44, 51) and a combination of both in one study (50). Biochemical biomarkers were compared to clinical NDO in 13 studies (24, 29, 47, 52–61), to brain MRI in one study (63) and to both in one study (62). In total, 2,846 individual cases were assessed, of which 908 with NIRS, 1163 with EEG and 903 with biochemical biomarkers.

As a clinical outcome measure, the Bayley Scales of Infant Development (BSID-II or III) was reported most frequently (*n* = 25). Other studies reported Pediatric Cerebral Performance Category (PCPC) (*n* = 2), Pediatric Stroke Outcome Measure (PSOM) (*n* = 1), age-appropriate IQ testing (Wechsler IQ scales or similar) (*n* = 5), Denver Developmental Screening test (DDST) (*n* = 1) or Vineland Adaptive Behavior Scale (VABS) (*n* = 3). Median age at clinical developmental testing was 15 (IQR 12–24) months.

For MRI-based outcomes, 10 studies used brain injury (stroke, hemorrhage and white matter injury) as outcome measures (32, 34–36, 38, 44, 50, 51, 62, 63), whereas others performed brain volumetry (33, 37, 39). Postoperative MRI was performed before discharge at varying timepoints.

All studies were executed in tertiary or quaternary hospital settings. Sixteen studies took place in the United States (24, 27, 28, 30, 31, 34, 36, 38, 45, 46, 48–50, 57, 59, 62) and 15 in European countries (25, 26, 29, 32, 33, 37, 39, 43, 44, 53, 55, 56, 60, 61, 63). The remaining 9 studies were conducted in Australia, New Zealand, China, Canada and Israel (35, 40–42, 47, 51, 52, 54, 58). No studies were conducted in Low- or Middle-Income Countries.

### 3.3 Results of included studies

An overview of the study characteristics, inclusion criteria, inclusion period and main results, can be found in Tables 1–3.

**Table 1:** Overview of studies reporting on perioperative NIRS^a^.

Author, Year	Type	Inclusion criteria	Intervention	N	Outcome and timing	Main results
Aly et al. (24)	Cohort	CPB^b^, < 1mo	cTOI^c^	54	BSID-II^d^ 6, 15 and 21 mo	Average cTOI at 60 min off CPB and 24 h postoperative was lower in patients with poor vs. good NDO^e^ 1% decrease in cTOI 24 h postoperative resulted in 7% increased risk in odds of a poor NDO
Carra et al. (25)	Cohort	<12y 2012–2015	cTOI	87	Wechsler^f^ 2y post-operative	Increased dose of cerebral desaturation in the 1st 12 h postoperative resulted in lower IQ
Hansen et al. (26)	Cohort	Stage 1 palliation 2006–2010	ScO_2_^g^	43	HAWIVA and KET-KID^h^ 5y	Preoperative ScO_2_ was correlated with NDO No association of NDO with duration of desaturation <40% or any ScO_2_ postoperative.
Hoffman et al. (27)	Cross-sectional	Stage 1 palliation 2002–2009	ScO_2_	21	VMI^i^, varied	Average ScO_2_ in the first 48 postoperative hours was lower in patients who demonstrated VMI scores <85 Patients with lower VMI had significantly more hours ScO_2_ < 45 and < 55% Patients without stroke and with any hourly ScO_2_ < 45% had lower VMI
Hoffman et al. (28)	Cross-sectional	CPB, <1y 2007–2014	ScO_2_	178	BSID-III^j^, varied	Difference between S_a_O_2_^k^ and ScO_2_ was strongly associated with motor performance but not with cognitive or language performance
Sanchez-De-Toledo et al. (29)	Cohort	CPB, <17y 2009–2010	ScO_2_	39	PCPC^l^ 2mo postoperative	ScO_2_ values were lower in patients with adverse NDO Patients with adverse NDO had significantly longer periods of ScO_2_ desaturations and AUC values below 20% of baseline
Simons et al. (30)	Cross-sectional	<12mo 2007	ScO_2_	26	BSID-III 24mo	None of the NIRS variables studied was associated with cognitive or fine motor scores in a multivariable model Patients with delayed expressive communication were more likely to have lowest ScO_2_ < 20% as compared with those without delay
Sood et al. (31)	Cross-sectional	<12mo 2007–2010	ScO_2_	31	BSID-III 24mo	Postoperative lowest ScO_2_ was predictive of cognitive and gross motor delay with thresholds <56 and < 49% respectively
Spaeder et al. (36)	Cross-sectional	CPB, <6w 2006–2012	cTOI	44	BSID-II 6, 15 and 21 mo Postoperative MRI^m^	Postoperative cTOI variability was lower in patients with poor NDO The ability of postoperative cTOI variability to discriminate PDI was fair. Its ability to discriminate MDI was good. A postoperative cTOI variability of less than 1.05 best predicted poor MDI There was no relationship between postoperative cTOI variability and MRI findings
De Silvestro et al. (37)	Cohort	CPB, <6w 2009–2020	ScO_2_	31	BSID-III 12mo Postoperative MRI	NDO did not differ in patients with or without cerebral desaturation Patients with cerebral desaturation <45% had larger relative lateral ventricle volume change per week Patients with >20% decrease in ScO_2_ had larger relative lateral ventricle volume change per week than patients without this decrease
Kussman et al. (38)	Cross-sectional	D-TGA, TOF, TA, VSD or AVSD^n^, <9mo 2001–2004	ScO_2_	89 40	BSID-II 1y Postoperative MRI	PDI significantly correlated with average and minimum ScO_2_ during the 60 min period following cessation of CPB No correlation was found between any intraoperative NIRS variable and MDI score Average ScO_2_ from post-induction to 60 min post-CPB was lower in subjects with hemosiderin depositions
Mueller et al. (39)	Cross-sectional	Stage 2 palliation 2012–2016	ScO_2_	19	BSID-III 25mo Postoperative MRI	No correlation between intraoperative cerebral NIRS parameters and NDO Positive correlation between the lowest measured intraoperative ScO_2_ and the lowest measured intracranial, total brain and white matter volume Intracranial volume was inversely correlated with the AUC of ScO_2_ < 45%
Claessens et al. (32)	Cohort	TGA, LVOTO, SVP^o^, <30d 2009–2012, 2016–2017	ScO_2_, FTOE^p^	74	Postoperative MRI	No relationship between ScO_2_ or FTOE with brain injury
Kelly et al. (33)	Case–control	<1y	ScO_2_	30	Preoperative MRI	ScO_2_ showed a modest correlation with whole brain gyrification index and grey matter volume
Lynch et al. (34)	Cohort	Stage 1 palliation 2008–2013	ScO_2_	37	Pre- and postoperative MRI	Patients with new or worsened postoperative periventricular leukomalacia tended to have lower preoperative ScO_2_
Zou et al. (35)	Cohort	<5y 2020–2021	ScO_2_, COPI^q^	65	Postoperative MRI	Magnitude of COPI and duration of abnormal COPI correlated with degree of brain injury

Inclusion period is mentioned whenever known. ^a^Near-Infrared Spectroscopy. ^b^Cardiopulmonary bypass. ^c^Cerebral Tissue Oxygenation Index, formula: TOI (%) = kO_2_Hb/(kO_2_Hb + kHHb) where k is the constant scattering contribution, O_2_Hb is oxygenated hemoglobin and HHb is reduced hemoglobin. ^d^Bayley Scales of Infant and Toddler Development, 2nd edition, consisting of Psychomotor Development Index (PDI) and Mental Development Index (MDI), domain scores have population mean 100(SD 15). ^e^Neurodevelopmental outcome. ^f^Wechsler preschool and Primary Scale of Intelligence (WPPSI-III-NL) for children 2.5–6 years, Wechsler Intelligence Scale for Children (WISC-III-NL) for children 6–16 years, mean 100(SD 15). ^g^Regional cerebral oxygen saturation. ^h^Hannover-Wechsler Intelligence Scale and Cognitive Development Test for Preschool-Age Children and Kognitiver Entwicklungstest für das Kindergartenalter. ^i^Beery-Buktenica Developmental Test of Visual Motor Integration, mean 100(SD 15). ^j^Bayley Scales of Infant and Toddler Development, 3rd edition, consisting of Cognitive, Language, Motor (gross and fine motor), Social–Emotional and Adaptive behavior domains, population mean 100(SD 15). ^k^Arterial oxygen saturation. ^l^Pediatric Cerebral Performance Category, 1 (normal function) – 6 (brain death). ^m^Magnetic Resonance Imaging. ^n^Dextrotransposition of the Great Arteries, Tetralogy of Fallot, Truncus Arteriosus and (Atrio)Ventricular Septal Defect. ^o^Transposition of the Great Arteries, Left Ventricle Outflow Tract Obstruction or Single Ventricle Physiology. ^p^Fractional Tissue Oxygen Extraction, formula: FTOE = (S_a_O_2_ – cTOI)/S_a_O_2_. ^q^Cerebral oximetry/pressure index, formula: COPI = (nΣxy − (Σx) (Σy))/((nΣx^2^ − (Σx)^2^)(nΣy^2^ − (Σy)^2^)) where n indicates the number of samples in each dataset, x indicates blood pressure, and y indicates ScO_2_. COPI > 0.3 indicates disturbed autoregulation.

**Table 2:** Overview of studies reporting on perioperative EEG^a^.

Author, Year	Type	Inclusion criteria	Intervention	N	Outcome and timing	Main results
Gui et al. (40)	Cross-sectional	<3mo 2015–2017	aEEG^a^	93	BSID-II^b^ 1y	MDI was significantly lower in children with absent postoperative SWC^c^ compared with immature postoperative SWC PDI was significantly lower in children with mild abnormal pre- or postoperative background pattern PDI was significantly lower in children with immature preoperative SWC
Gunn et al. (41)	Cohort	<2mo 2005–2008	aEEG	125	BSID-III^d^ 2y	No association between perioperative seizures or preoperative background to NDO^e^ Prolonged recovery to continuous background was associated with lower cognitive and motor scores Delayed recovery of SWC was associated with lower cognitive scores
Gunn et al. (42)	Cohort	Stage 1 palliation 2005–2008	aEEG	25	BSID-III 2y	No association between perioperative seizures and NDO Recovery to a continuous background within 48 h was associated with a 14-point increase in motor score
Latal et al. (43)	Cohort	CPB^f^, <3mo 2006–2009	aEEG	50	BSID-II 1y IQ testing^g^ 4y	Postoperative seizures were associated with lower MDI Postoperatively persistent discontinuous background was associated with lower MDI and lower IQ Delayed recovery of SWC was associated with lower IQ
Claessens et al. (44)	Cross-sectional	Neonatal surgery^h^ 2009–2019	aEEG	73	Postoperative MRI	Abnormal postoperative background pattern was more common in neonates with new postoperative injury than without brain injury Neonates with postoperative seizures were at higher risk for new brain injury
Gaynor et al. (45)	Cohort	CPB, <6mo 2001–2003	cEEG^a^	114	BSID-II 1y	Frontal onset seizures were predictive of lower MDI scores compared non–frontal-onset seizures
Gaynor et al. (46)	Cohort	CPB, <6mo, 2001–2003	cEEG	132	Multiple^i^, 4y	No association of seizures with cognition, motor or language outcomes, but increased prevalence of executive dysfunction
Robertson et al. (47)	Cohort	CPB, <4mo 1999–2001	cEEG	35	BSID-II 1y post-operative	No difference in NDO between patients with abnormal and normal EEG
Seltzer et al. (48)	Cohort	<30d	cEEG	21	VABS^j^ 5y	Infants who developed an isoelectric state had significantly lower communication subscores Isoelectric state time > 90 min was associated with lower NDO Longer duration of the isoelectric state was associated with lower NDO
Vaughan et al. (49)	Cross-sectional	<60d	cEEG 2010–2021	76	BSID-III 9,18,24, 30mo	Preoperative diffuse abnormalities were associated with lower cognitive scores Cognitive scores were lower in patients with preoperative waveform discontinuity or postoperative absence of behavioral state change Preoperative continuous waveforms were associated with higher cognitive scores, whereas postoperative continuous waveforms were associated with higher fine motor scores Preoperative synchrony was associated with higher fine motor scores in the 1^st^ year postoperative
Rappaport et al. (50)	Cohort	D-TGA^k^	cEEG 1988–1992	155	BSID-II 1y Postoperative MRI	Children with seizures had lower PDI scores than children without seizures Children with EEG seizures were more likely to have MRI abnormalities
Lin et al. (51)	Cohort	<3y	cEEG 2019–2021	264	Postoperative MRI	EEG abnormalities (except spikes/sharp waves and delta brushes) were associated with degree of brain injury Patients not recovering to the normal background and SWC by 48 h postoperative had worse degree of injury Longer duration of isoelectric EEG was associated with more severe postoperative degree of brain injury

Inclusion period is given whenever known. ^a^Amplitude-integrated Electroencephalography (aEEG) and continuous EEG (cEEG). ^b^Bayley Scales of Infant and Toddler Development, 2nd edition, consisting of Psychomotor Development Index (PDI) and Mental Development Index (MDI), domain scores have population mean 100(SD 15). ^c^Sleep-wake cycling. ^d^Bayley Scales of Infant and Toddler Development, 3rd edition, consisting of Cognitive, Language, Motor (gross and fine motor), Social–Emotional and Adaptive behavior domains, population mean 100(SD 15). ^e^Neurodevelopmental outcome. ^f^Cardiopulmonary Bypass. ^g^IQ testing either by Wechsler Preschool and Primary Scale of Intelligence-3 (*n* = 29), Snijders Omen Test of Intelligence (*n* = 18) or Kaufman-ABC-II (*n* = 1). ^h^Critical congenital heart disease requiring neonatal surgery, defined as biventricular with or without aortic arch obstruction, or single ventricle physiology.^i^ Comprehensive evaluation with Wechsler Preschool and Primary Scale of Intelligence-3, Preschool Language Test-4, Wide Range assessment of Visual Motor abilities, Developmental test of Visual Motor Integration, NeuroPsychology Attention/Executive functions Domain Score, Child Behavior Checklist, ^j^Vineland Adaptive Behavior Scale, z-value mean 0 (SD 1.0). ^k^Dextrotransposition of the Great Arteries.

**Table 3:** Overview of studies reporting on perioperative biochemical biomarkers.

Author, Year	Type	Inclusion	Intervention	N	Outcome and timing	Main results
Aly et al. (24)	Cohort	CPB^a^, < 1mo	Lactate	54	BSID-II^b^ 6, 15, 21mo	Patients with mortality or poor NDO^c^ had a higher lactate concentration compared with survivors with good NDO at 60 min off CPB and at 24 h postoperative
Bar-Yosef et al. (52)	Case–control	<4y 2015–2017	S100B	75	PSOM^d^ before discharge	S100B z-scores >3SD 6 h post-surgery predicted new neurological deficit (at least 1 point increase in PSOM) S100B z-scores before surgery were significantly associated with new neurological deficit
Cañizo Vázquez et al. (53)	Cohort	<7 m 2017–2019	8-iso-PGF2, S100B	44	BSID-III^e^/ VABS^f^ 24mo	Patients with abnormal NDO had higher levels of 8-iso-PGF2 at 24 h postoperative, but without enough power to predict abnormal NDO S100B levels at 72 h postoperative were a strong predictor of abnormal NDO
Cheung et al. (54)	Cohort	CPB, <6w 1996–1999	Lactate	67	BSID-II 18-24mo	Lactate >6 mmol/L on day 1 postoperative predicted adverse outcome (death or poor NDO)
Chiperi et al. (55)	Cohort	<5y 2022–2023	GFAP, BDNF, S100B, NSE	42	DDST II^g^ 4–6mo postoperative	GFAP predicted abnormal NDO in cyanotic patients
Gessler et al. (56)	Cohort	CPB, 3mo-7y	IL-6, IL-8	31	BSID-II 6mo post-operative	Plasma levels of IL-6 at 3 h post-CPB significantly predicted NDO
Graham et al. (57)	Cross-sectional	CPB, <1mo 2012–2017	GFAP	97	BSID-III 12mo	GFAP at cessation of CPB was independently associated with motor composite scores Cognitive and language composite scores were not independently associated with GFAP levels
Gunn et al. (58)	Cohort	<2mo 2005–2008	S100B, lactate	130	BSID-III 2y	Higher lactate at 24 h was associated with impaired NDO Elevated S100B at 48 h predicted motor outcome
Robertson et al. (47)	Cohort	CPB, 16d-4mo 1999–2001	S100B	35	BSID-II 1y post-operative	Elevated levels of S100B immediately postoperative or 24 h after CPB did not predict NDO
Sanchez-De-Toledo et al. (29)	Cohort	CPB, < 17y 2009–2010	NSE, S100B, GFAP, BDNF	39	PCPC^h^ 12mo post-operative	No significant differences in serum neuromarkers at baseline, at the end of CPB or 16 h postoperative between groups with different PCPC scores
Trakas et al. (59)	Cohort	CPB, <30d	NSE, S100B	18	PCPC before discharge	No significant association of postoperative neuronal biomarker levels and PCPC score at discharge
Vedovelli et al. (60)	Cohort	CPB, <3y 2014–2016	GFAP	45	VABS 18mo	Communication IQ was predicted significantly by the highest measured GFAP
Vergine et al. (61)	Cross-sectional	CPB, <5y 2010–2017	GFAP	38	NDI^i^ 1y	Maximum GFAP level was significantly associated with NDI No association of GFAP levels with IQ
De Ferranti et al. (62)	Cross-sectional	D-TGA^j^ 1988–1992	Glucose	155	BSID-II 1y Postoperative MRI	NDO was not related to perioperative glucose levels MRI findings were not related to perioperative glucose levels
Jungner et al. (63)	Cohort	CPB, <30d	GFAP, NfL, Tau	33	Postoperative MRI	The relative increase in plasma Tau from preoperative concentrations until postoperative day 2 was significantly higher in infants with postoperative white matter injury

^a^Cardiopulmonary Bypass. ^b^Bayley Scales of Infant and Toddler Development, 2nd edition, consisting of Psychomotor Development Index (PDI) and Mental Development Index (MDI), domain scores have population mean 100(SD 15). ^c^Neurodevelopmental outcome. ^d^Pediatric Stroke Outcome Measure, scores 0 (no deficit) – 10 (maximum deficit). ^e^Bayley Scales of Infant and Toddler Development, 3rd edition, consisting of Cognitive, Language, Motor (gross and fine motor), Social–Emotional and Adaptive behavior domains, population mean 100(SD 15). ^f^Vineland Adaptive Behavior Scale, z-value mean 0 (SD 1.0). ^g^Denver Developmental Screening test II, consisting of personal-social behavior, fine-motor adaptive function, gross motor function and language domains, adapted to age. ^h^Pediatric Cerebral Performance Category, 1 (normal function) – 6 (brain death). ^i^Neurodevelopmental Index. Altered NDI if ≥2 severe impairments at neuropsychological tasks (<2SD) on the NEPSY-II attention, executive function, social skills, theory of mind or emotional recognition testing, an IQ <85 on the Wechsler Preschool and Primary Scale of Intelligence III test or the Wechsler Intelligence Scale for Children IV and/or a clinically relevant score on the Child Behavior Checklist/Conner’s Parent Rating Scales (>65 T scores). ^j^Dextrotransposition of the Great Arteries.

#### 3.3.1 Association of NIRS with neurological outcome

Cerebral oximetry using NIRS is a non-invasive monitoring technique based on detection of hemoglobin oxygenation using near-infrared light, from which parameters such as Cerebral Oxygen Saturation (ScO_2_), Cerebral Tissue Oxygenation Index (cTOI) and Fractional Tissue Oxygen Extraction (FTOE) can be derived. Combining NIRS parameters with heart rate or blood pressure measurements allows for the measurement of cerebral autoregulation, for example by calculating Cerebral Oximetry/Pressure Index (COPI), as COPI >0.3 indicates disturbed autoregulation.

##### 3.3.1.1 Association with clinical NDO

Twelve articles examined the association of NIRS variables with clinical NDO (Table 1).

Pre-operative ScO_2_ before stage 1 palliation was associated with cognitive scores in the report by Hansen et al. (26). Intraoperatively, a retrospective study of children with biventricular CHD without aortic arch obstruction, found a correlation of intraoperative ScO_2_ immediately post-CPB surgery with Psychomotor Developmental Index (PDI) but not with Mental Developmental Index (MDI) subscores of the BSID-II (38). In contrast, Mueller et al. did not report any associations between NIRS values during stage 2 palliation and BSID scores (39).

Postoperatively, cTOI after neonatal CPB surgery significantly predicted mortality and poor BSID scores (24), and postoperative cTOI variability was able to discriminate between poor and good BSID (36). Hoffman et al. reported associations of average postoperative ScO_2_ with NDO after stage 1 palliation (27). Postoperative cerebral desaturation predicted poorer NDO in three mixed CHD populations (25, 28, 31). Conversely, Hansen et al. did not report any association of NDO with NIRS after stage 1 palliation (26).

Some studies reported on findings in the entire perioperative period rather than specific timepoints. Both perioperative ScO_2_ and cerebral desaturation were associated with NDO in children undergoing CPB surgery in childhood (29). Comparable results on cerebral desaturation were reported in children below 1 year of age, although they only reached significance in the expressive communication BSID subscore (30). Hoffman et al. described an association of cerebral desaturation and the difference between arterial and cerebral oxygenation with NDO in two retrospective cohorts (27, 28). Contrarily, De Silvestro et al. did not find an association between cerebral desaturation around neonatal CPB surgery and BSID scores at 1 year (37).

##### 3.3.1.2 Association with brain MRI

The eight studies reporting on the association of NIRS and MRI are summarized in Table 1.

Pre-operative ScO_2_ values were associated with brain injury after stage 1 palliation in one study (34). Two studies researched intraoperative ScO2: one found an association with both lower brain volumetry in hypoplastic left heart syndrome (HLHS) and the other with brain injury in a mixed cohort (38, 39). Spaeder et al. found no relationship between postoperative cTOI variability and MRI findings (36).

Again, perioperative timepoints were not always specified. An association between perioperative ScO_2_ and lower brain volumes was found in a mixed cohort by Kelly et al. (33), whereas cerebral desaturation significantly correlated with lower volumetry in the report on HLHS by Mueller et al. and the study on neonatal CPB surgery by De Silvestro et al. (37, 39). Zou et al. found that the magnitude and duration of abnormal COPI correlated with degree of brain injury (35). On the other hand, Claessens et al. did not find an association between ScO_2_ or FTOE surrounding CPB surgery and brain injury (32).

#### 3.3.2 Association of EEG with neurological outcome

EEG electrodes are placed on the scalp for detection of the spontaneous electrical activity of the brain. The EEG can be utilized for different indications, such as the detection of subclinical, electrographic seizures and the review of background activity and sleep–wake cycling. cEEG uses the full array of scalp electrodes according to the international 10–20 system (modified for neonates) and provides detailed information on the temporospatial occurrence of electrical potentials. In contrast, aEEG is a simplified method using a more limited number of electrodes (up to 4) and provides a time-compressed signal based on the amplitude of the electrographic signal. It is useful in background detection and detection of sleep–wake cycling, and has its value in seizure detection although with less sensitivity compared to cEEG, and with loss of temporospatial information.

For this review, we included studies reporting on both cEEG and aEEG, as the outcomes of interest should be detectable on both modalities.

##### 3.3.2.1 Association with clinical NDO

Ten studies examined the association of EEG variables with clinical NDO (Table 2). Four studies used aEEG and six used cEEG.

Using aEEG, Latal et al. showed lower cognitive BSID subscores in patients with electrographic seizures after CPB surgery before 3 months of age (43) whereas Gunn et al. did not report any association between perioperative seizures and BSID scores (41, 42).

Using cEEG, The Boston Circulatory Arrest group showed that electrographic seizures during the arterial switch operation were associated with lower psychomotor BSID subscores at 1 year of age (50). Gaynor et al. found lower cognitive BSID scores with frontal-onset seizures at 1 year of age in children undergoing CPB surgery before 6 months (45) and executive dysfunction at 4 years of age in patients experiencing perioperative electrographic seizures (46). Robertson et al. did not find any association between seizures measured with cEEG and BSID scores (47).

Both Gui et al. and Vaughan et al. described an association of preoperative background pattern with BSID scores (40, 49), whereas two others did not confirm this association (41, 47). All four studies included mixed CHD types. Postoperatively, an abnormal background pattern was consistently associated with poor NDO in 5 studies: in particular, delayed recovery of sleep–wake cycling, duration of the isoelectric state and prolonged discontinuity were associated with poor NDO (40–43, 48).

##### 3.3.2.2 Association with brain MRI

Three studies report on the association of postoperative EEG variables with brain injury on MRI (Table 2). Claessens et al. describe both postoperative abnormal background and ictal discharges on aEEG as risk factors for new-onset brain injury (44). The association with electrographic seizures on cEEG is also reported in the Boston Circulatory Arrest cohort (50). In addition, Lin et al. found that electrographic abnormalities seen on cEEG were associated with brain injury, with patients with longer isoelectric traces or not recovering to normal background and sleep–wake cycling by 48 h having worse degree of injury (51).

#### 3.3.3 Association of biochemical biomarkers with clinical NDO or brain MRI

A large variety of biochemical biomarkers of brain injury are being studied in relation to neurological outcome. The 15 included studies are summarized in Table 3. S100 calcium-binding protein B (S100B) was most studied in association with NDO. Of the seven studies reporting this outcome, four did not find anycorrelation (29, 47, 55, 59). One study found that elevated S100B at 48 h after CHD surgery before 2 months of age predicted BSID motor outcome at 2 years, and another found associations between S100B at 72 h postoperative and BSID at 2 years (53, 58). In addition, In children undergoing CHD surgery before 4 years of age, elevated S100B was associated with new neurological deficit upon discharge (52).

Six studies studied glial fibrillary acidic protein (GFAP) as a predictive biomarker. Chiperi et al. report its predictive ability for DDST scores in cyanotic patients undergoing surgery before 5 years of age (55), while Graham et al. found an association with BSID motor but not with cognitive and language scores in infants undergoing CPB surgery in their first month (57). The highest measured GFAP was able to predict communication intelligence quotient using the VABS at 18 months in the study by Vedovelli et al. (60). In contrast, Vergine et al. did not find an association of GFAP levels during CPB surgery with cognitive abilities, although did relate to the composite neurodevelopmental scoring system used in their study (61). Sanchez-De-Toledo et al. did not did not report significant associations with PCPC scores 12 months after CPB surgery in childhood (29). In addition to GFAP, the report by Jungner et al. examined Neurofilament light polypeptide (NfL) and Tau and described that patients with postoperative white matter injury on brain MRI had a significantly higher increase in plasma Tau levels, but no associations with GFAP or NfL were found (63).

PCPC scores at discharge were not related to levels of neuron-specific enolase (NSE) or brain-derived neurotropic factor (BDNF) in infants undergoing CPB surgery before 30 days, and neither were NSE and PCPC scores at 12 months postoperative in pediatric CPB surgery for CHD (29, 59). Another study reporting DDST scores 4–6 months after surgery, did not find an association with NSE or BDNF levels during CHD surgery (55). Three studies reported that perioperative lactate elevations significantly predicted poor BSID scores (24, 58, 65). Cañizo Vàzquez et al. reported increased levels of 8-iso-prostaglandin F2α (8-iso-PGF2), a urinary biomarker for oxidative stress, in patients with abnormal BSID or VABS scores. In an article by Gessler et al., plasma IL-6 at 3 h post-CPB significantly predicted NDO (56). Lastly, one article explored perioperative glucose levels during the arterial switch operation, but found no association with clinical NDO or MRI (62).

### 3.4 Risk of bias

**Figure 2:**
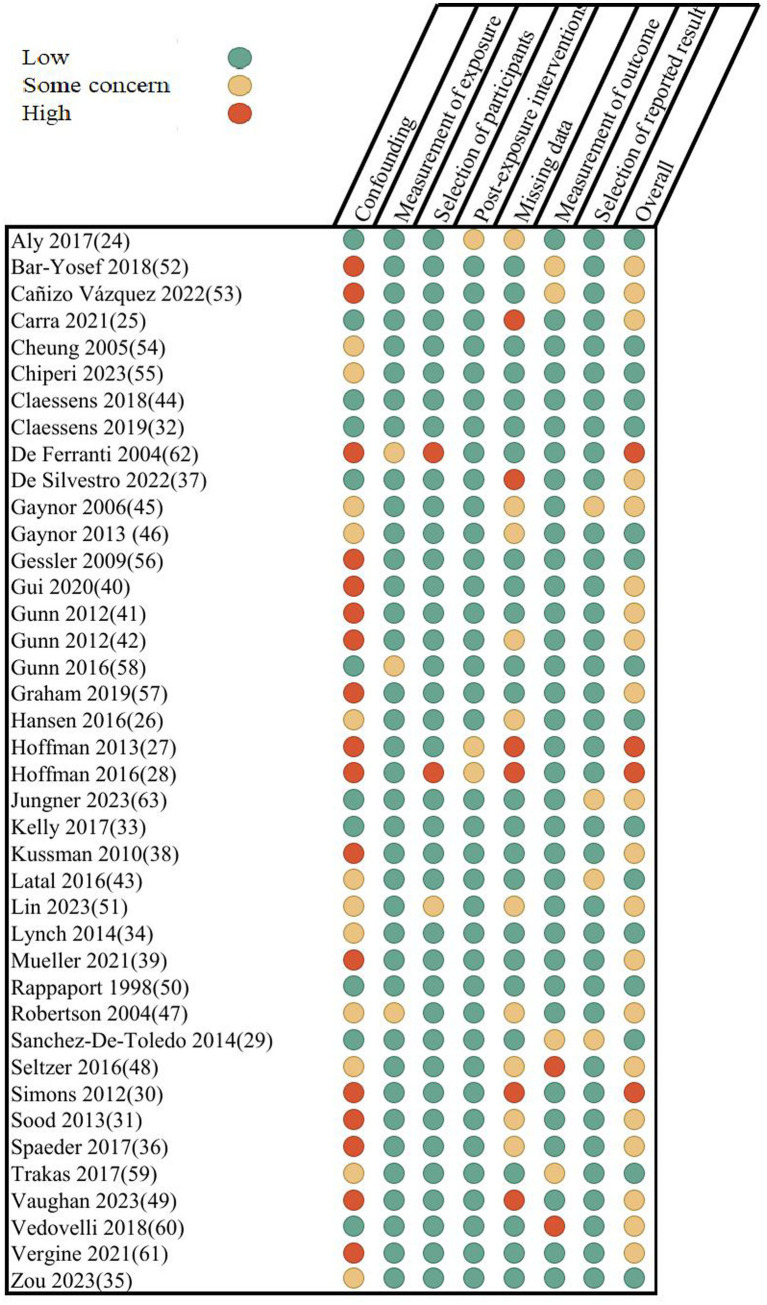
ROBINS-E tool for risk of bias assessment.

Risk of bias across studies was assessed using the ROBINS-E tool. Results are presented in Figure 2. Risk of bias was overall low to moderate, with concerns mostly due to the risk of confounding, inherent to the observational study design. The risk of selection of reported results seemed low overall, given that the reported outcomes were consistent with preregistered protocols and also non-significant data was reported.

Four studies were judged to have high risk of bias due to the large proportion of missing data (27, 28, 30, 49). Additionally, the studies by Hoffman et al. (27, 28) selected patients based on referral for neurodevelopmental testing (possibly due to suspicion of developmental impairment). Two studies also included patients with genetic syndromes, likely influencing neurodevelopmental outcome scores (28, 46). Two studies (48, 60) posed some concern for bias because their outcomes were (in part) based on subjective parent-based questionnaires, although this was accounted for by the use of a validated scoring system (VABS).

### 3.5 Quality of evidence

**Figure 3:**
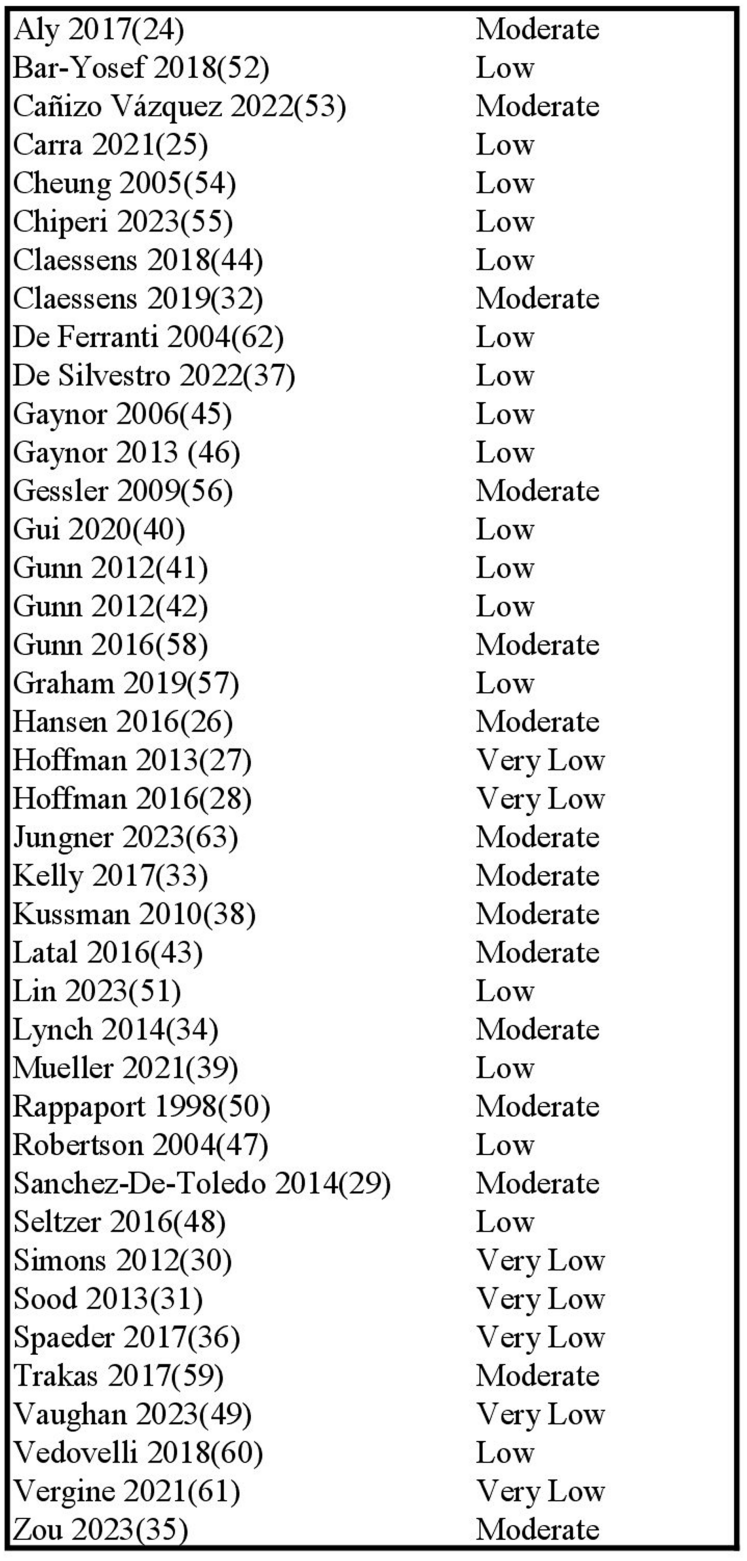
GRADE assessment of quality of evidence.

We rated the quality of evidence using the GRADE approach (23), results are presented in Figure 3. The included reports provided mostly low-quality evidence due to their observational nature. Studies were upgraded to moderate-level evidence if they had strong designs, were well conducted, and had few major flaws. Studies were downgraded if there was risk of bias or inconsistency in results.

## 4 Discussion

This review article summarizes existing data regarding neuromonitoring using perioperative NIRS, EEG, and serum brain biomarkers and their association with neurodevelopmental outcome, brain maturation and brain injury on MRI after surgery for congenital heart disease. This type of data is potentially valuable in risk stratification, in light of recent guidelines supporting neurodevelopmental follow-up for certain subgroups of CHD patients, which is resource-intensive and of variable availability.

First, the majority of studies evaluating NIRS suggest an association between cerebral oxygenation measurements (cTOI, ScO_2_, cerebral desaturation) and the pre-specified neurological outcomes (either clinical or neuroimaging). It is important to note however, that there was large variability in the thresholds to define cerebral desaturation, as well as the exposure measures (binary outcome versus time below a certain threshold). Interestingly, disturbed cerebral autoregulation seemed to correlate with neurological outcome.

Secondly, electrographic seizures, either measured with cEEG or aEEG, were independently associated with poorer neurological outcome in the majority of reports and patients with prolonged abnormal background patterns or delayed return of sleep–wake cycling, were at risk for adverse outcome.

Thirdly, the predictive ability of biomarkers such as S100B and GFAP is not sufficiently convincing. Postoperatively increased lactate however, was associated with neurodevelopment, in addition to its known associations with short-term adverse outcomes (66, 67). According to the included studies, significant elevation >4–6 mmol/L or persistent elevation beyond 24 h seemed predictive of outcome. This is an important finding as most cardiac centers already routinely measure lactate levels as an indicator of the hemodynamic and metabolic condition of the patient. For the other neuronal markers mentioned in this study, evidence is too sparse to draw a sound conclusion. The ability to draw stronger conclusions and perform meta-analyses was limited by the inconsistency and heterogeneity of the findings. This review provides an update to existing literature by comprehensively and systematically summarizing available data on perioperative neuromonitoring and its association with outcomes. These findings build on recent publications showing that patients with CHD show abnormal electroencephalographic activity even in the preoperative period (68, 69), and impaired perioperative autoregulation which cannot simply be explained by differences in blood pressure (70). In addition, the perioperative period is characterized by significant hemodynamic disturbances and might require even more from the autoregulatory mechanisms of the brain. Moreover, new information on the disrupted autonomic regulation and altered circadian rhythms in CHD is arising.

Knowledge on these neuromonitoring modalities is steadily expanding in different fields of medicine, and it might be possible to extrapolate, without generalizing to the unique group of neonates with CHD. Cerebral NIRS monitoring has been utilized to guide treatment in the preterm population as a predictor of neurodevelopmental impairment in preterm neonates (71, 72), although in a large randomized trial on treatment guided by cerebral oximetry, no difference in serious adverse events was found (73). Secondly, in neonatal and pediatric patients undergoing extracorporeal life support (ECLS), the prognostic value of cerebral oximetry on neurodevelopment was demonstrated (74, 75), and a pilot study highlighted the importance of impaired cerebral autoregulation in patients with acute neurological events (76). aEEG and cEEG are widely used in preterm babies as a biomarker of brain injury, as a modality to monitor brain maturation and as a prognostic tool for subsequent neurodevelopment (77, 78). Fogtmann et al. performed a systematic review on this topic which demonstrated the good predictive value of EEG for NDO after prematurity (79). In hypoxic–ischemic encephalopathy, the prognostic value of NIRS and EEG has repeatedly been demonstrated (80, 81). Specifically, studies showed that that prolonged abnormal background on EEG was associated with impaired neurodevelopment (82–84), and seizure burden was predictive of neurodevelopmental outcome (85–87). EEG seizure burden and asymmetric EEG background correlated with brain injury in two pediatric ECLS populations (88, 89), while background abnormalities predicted poor NDO with good specificity (90). The utility of different neuromonitoring modalities in ECLS is summarized in the review by Felling et al. (91). The information extrapolated from these patient groups could aid in the comprehension of underlying autoregulation mechanisms in patients with CHD. The field of biochemical neurological biomarkers has predominantly been studied in adult settings and evidence from pediatric populations is only recently emerging. For example, a wide array of biomarkers is being assessed for outcome prediction after ischemic stroke in adults (92, 93). In pediatric traumatic brain injury, S100B levels correlated to the extent of brain injury (94). In pediatric ECLS patients, elevation of plasma brain injury biomarkers was associated with unfavorable NDO and brain imaging abnormalities (95). Even though the underlying conditions differ from CHD, they face many similar difficulties concerning hemodynamic disturbances, inflammation, altered cerebral perfusion and reperfusion injury (96), which makes it plausible that neuromonitoring modalities used in these clinical settings could potentially be of use in the setting of CHD. In addition, Chiperi et al. (97) recently published a systematic review on the use of biochemical biomarkers in pediatric CHD surgery: they reported poor predictive value of NSE and BDNF and conflicting results on S100B, but suggested GFAP as a possible biomarker for brain injury.

### 4.1 Limitations

The results of this analysis should be interpreted with necessary caution. Whereas the single studies are overall of good quality, although observational in design, the studies included relatively small and diverse groups of patients, with different cardiac diagnoses, age ranges and treatment strategies. Even though most studies only included patients with normal neurologic exams before surgery, pre-existing comorbidities may be unaccounted for. Studies were conducted in different epochs causing differences in surgical and medical management strategies. Additionally, there was large variability in reported NIRS variables across studies, rendering a quantitative meta-analysis impossible. A possible explanation is that patients with varying CHD types have altered baseline ScO_2_ from the healthy population, making current definitions of cerebral desaturation (typically ScO_2_ < 45 or < 65%) less relevant (98). In future analyses, a relative decrease in ScO_2_ from baseline may be more valuable than absolute values. The duration of EEG monitoring was variable (pre- vs. postoperative, duration of postoperative monitoring) and authors did not control for the effects of sedative medications on EEG traces, possibly introducing bias as the most unstable patients likely received more suppressive medication.

There was large variability in timing of outcome measurements, which were mostly short-term, and instruments for clinical assessment of neurological outcome focused on different neurodevelopmental domains. For example, postoperative PCPC or PSOM may indicate global neurological outcome before discharge, whereas comprehensive neurodevelopmental assessment at preschool age is the preferred method for detailed evaluation of long-term neurological outcomes. No studies were conducted in Low or Middle-Income Countries, limiting generalizability to these countries.

Should we aim to establish guidelines surrounding effective perioperative neuromonitoring, there is a need for larger, more definitive studies with increased consistency in the application of these modalities and definition of clinically useful outcomes. In addition, patient-specific (e.g., socio-economic status, (epi)genetics) and medical factors (e.g., surgical technique, length of stay, ventilation time, sedative use) should be considered as they impact the cerebral compensatory mechanisms and might better explain the variability in neurological outcome in this population. Longer-term data using outcome measures encompassing different neurodevelopmental domains (cognitive, psychomotor, visuospatial, executive functioning) is necessary to more accurately predict the long-term prognosis of these patients.

### 4.2 Conclusion

To conclude, there is some evidence indicating an association between perioperative non-invasive neuromonitoring modalities and neurological outcome in the CHD population, most convincingly cerebral desaturation and autoregulation measured with NIRS, electrographic seizures, and prolonged background abnormalities on EEG, and elevated lactate in the perioperative period. These results should be interpreted with caution however, and in further research, the standardization of perioperative monitoring application and outcome determination is necessary before clinical implementation of these strategies for neurological prognostication.

## Data Availability

The original contributions presented in the study are included in the article/Supplementary material, further inquiries can be directed to the corresponding author.
