# Supplementary material for: Neuromonitoring modalities predicting neurological impairment in pediatric congenital heart disease: a systematic review
Source: Front Neurol. 2024 Dec 18;15:1502762. doi: 10.3389/fneur.2024.1502762 (PMC11688251; doi:10.3389/fneur.2024.1502762)
Supplement: Supplementary file 1 [file Data_Sheet_1.docx]

**SUPPLEMENTARY MATERIALS**

**1 Search Strategy**

- 1. ***Medline***

("Heart Defects, Congenital"[Mesh] OR "congenital heart defect*"[tiab] OR "congenital heart disease*"[tiab] OR "congenital cardi*"[tiab] OR "congenital heart disorder*"[tiab] OR "heart abnormalit*"[tiab] OR "heart malformation*"[tiab] OR "malformation* of heart*"[tiab] OR "malformation* of the heart"[tiab] OR "cyanotic heart disease*"[tiab] OR "cyanotic cardi*"[tiab] OR "cyanotic heart defect*"[tiab] OR "cyanotic heart disorder"[tiab:~0] OR "cyanotic heart disorders"[tiab:~0] OR "pediatric heart disease*" [tiab] OR "paediatric heart disease*" [tiab] OR "pediatric cardi*"[tiab] OR "aortic coarctation"[tiab] OR "coarctation of the aorta"[tiab] OR "aorta coarctation"[tiab] OR "hypoplastic aortic arch"[tiab] OR "interrupted aortic arch"[tiab] OR "aortic arch interruption"[tiab] OR "coarctatio aortae"[tiab] OR "aortico ventricular tunnel"[tiab:~1] OR "aortico ventricular tunnels"[tiab:~1] OR "aorticoventricular tunnel*"[tiab] OR "aortoventricular tunnel*"[tiab] OR "aortic ventricular tunnel*"[tiab] OR "aorto ventricular tunnel*"[tiab] OR "cor triatriatum"[tiab] OR "subdivided left atrium"[tiab] OR "triatrial heart*"[tiab] OR ((congenital[tiab]) AND ("coronary vessel anomal*"[tiab] OR "coronary vessel malformation"[tiab:~0] OR "coronary vessel malformations"[tiab:~0])) OR "crisscross heart*"[tiab] OR "criss cross heart*"[tiab] OR "Situs Inversus"[Mesh] OR "situs inversus"[tiab] OR "dextrocardia"[tiab] OR "Kartagener"[tiab] OR "ciliary dyskinesia"[tiab] OR "levocardia"[tiab] OR "patent ductus arteriosus"[tiab] OR "patency of the ductus arteriosus"[tiab] OR "patent ductus botalli"[tiab] OR "open ductus botalli"[tiab] OR "ductus arteriosus persisten*"[tiab] OR "persistent ductus arteriosus "[tiab] OR "persistent ductus botalli"[tiab:~0] OR "ductus arteriosus patency"[tiab] OR "ductus botalli patency"[tiab:~0] OR "patent foramen ovale"[tiab] OR "Ebstein*"[tiab] OR "Ectopia cordis"[tiab] OR "ectocardia"[tiab] OR "ectopic heart"[tiab] OR "exocardia"[tiab] OR "cardiac ectop*"[tiab] OR "Eisenmenger*"[tiab] OR "Heart septal defect*"[tiab] OR "Heart septum defect*"[tiab] OR "cardiac septum defect*"[tiab] OR "cardiac septal defect*"[tiab] OR "Aortopulmonary Septal Defect*"[tiab] OR "Aortopulmonary septum Defect*"[tiab] OR "aorticopulmonary Septal Defect*"[tiab] OR "aorticopulmonary septum defect"[tiab:~0] OR "truncus arteriosus"[tiab] OR "endocardial cushion defect*"[tiab] OR "atrioventricular canal"[tiab] OR "atrial septal defect*"[tiab] OR "atrial septum defect*"[tiab] OR "persistent ostium primum"[tiab] OR "patent foramen ovale"[tiab] OR "patent oval foramen"[tiab] OR "lutembacher"[tiab] OR "ventricular septal defect*"[tiab] OR "ventricular septum defect*"[tiab] OR "intraventricular septal defect*"[tiab] OR "intraventricular septum defect*"[tiab] OR "double outlet right ventricle"[tiab] OR "Taussig-Bing Anomaly"[tiab] OR "atrioventricular septal defect*"[tiab] OR "atrioventricular septum defect*"[tiab] OR "atrio ventricular septal defect*"[tiab] OR "atrio ventricular septum defect"[tiab:~0] OR "atrio ventricular septum defects"[tiab:~0] OR "heterotaxy syndrome*"[tiab] OR "visceral heterotax*"[tiab] OR "situs ambiguus viscerum"[tiab] OR "atrial isomerism*"[tiab] OR "Ivemark Syndrome"[tiab] OR "HLHS"[tiab] OR "hypoplastic left heart"[tiab] OR "hypoplastic right heart"[tiab] OR "single ventricle"[tiab] OR "univentricular heart"[tiab] OR "monoventricular heart"[tiab] OR "non compaction ventricular myocardium"[tiab:~2] OR "noncompaction ventricular myocardium"[tiab:~2] OR "Fallots"[tiab] OR "Fallot"[tiab] OR "transposition of the great arter*"[tiab] OR "transposition of the great vessels"[tiab] OR "great vessels transposition*"[tiab] OR "great arteries transposition*"[tiab] OR "transposition of great arteries"[tiab:~1] OR "transposition of great vessels"[tiab] OR "levotransposition*"[tiab] OR "dextrotransposition*"[tiab] OR (("congenital"[tiab] OR "pediatric"[tiab] OR "paediatric"[tiab]) AND ("Cardiomyopathies"[Mesh:NoExp])) OR "congenital cardiomyopathy"[tiab:~1] OR "congenital cardiomyopathies"[tiab:~1] OR "pediatric cardiomyopathy"[tiab:~1] OR "paediatric cardiomyopathies"[tiab:~1] OR "paediatric cardiomyopathy"[tiab:~1] OR "pediatric cardiomyopathies"[tiab:~1] OR "arrhythmogenic right ventricular cardiomyopath*"[tiab] OR "arrhythmogenic right ventricular dysplasia"[tiab] OR "Scimitar Syndrome"[Mesh] OR "anomalous pulmonary venous return"[tiab] OR "Scimitar"[tiab] OR "TAPVR"[tiab] OR "pulmonary venous return anomal*"[tiab] OR "PAPVR"[tiab] OR "Persistent Left Superior Vena Cava"[Mesh] OR "left superior vena cava"[tiab] OR "left-sided superior vena cava"[tiab] OR "Bilateral superior vena cava"[tiab] OR "Bilateral superior caval veins"[tiab] OR "superior vena cava duplication"[tiab] OR "bilateral SVC"[tiab] OR "SVC duplication*"[tiab] OR "persistent superior vena cava"[tiab] OR "Vascular Ring"[Mesh] OR "vascular ring*"[tiab] OR "pulmonary artery sling"[tiab] OR "double aortic arch"[tiab] OR "right aortic arch"[tiab] OR (("congenital"[tiab] OR "pediatric"[tiab] OR "paediatric"[tiab]) AND ("Heart Valve Diseases"[Mesh] OR "heart valve disease*"[tiab] OR "heart valvular disease*"[tiab] OR "valvular heart disease*"[tiab] OR "aortic valve disease*"[tiab] OR "aortic heart disease*"[tiab] OR "aortic valvular heart disease*"[tiab] OR "aortic valve insufficiency"[tiab] OR "aortic valve incompetence"[tiab] OR "aortic regurgitation"[tiab] OR "aortic valve regurgitation"[tiab] OR "aortic incompetence"[tiab] OR "aortic insufficiency"[tiab] OR "aortic valve prolapse*"[tiab] OR "aortic valve stenos*"[tiab] OR "aortic stenos*"[tiab] OR "mitral valve prolapse*"[tiab] OR "tricuspid valve prolapse"[tiab] OR "mitral valve disease"[tiab] OR "mitral valve insufficiency"[tiab] OR "mitral valve incompetence"[tiab] OR "mitral regurgitation"[tiab] OR "mitral valve regurgitation"[tiab] OR "mitral incompetence"[tiab] OR "mitral insufficiency"[tiab] OR "mitral valve stenos*"[tiab] OR "mitral stenos*"[tiab] OR "pulmonary valve stenos*"[tiab] OR "pulmonary stenos*"[tiab] OR "pulmonic stenos*"[tiab] OR "pulmonary valve disease"[tiab] OR "pulmonary valve regurgitation"[tiab] OR "pulmonary valve insufficiency"[tiab] OR "pulmonary valve incompetence"[tiab] OR "pulmonary regurgitation"[tiab] OR "pulmonary insufficiency"[tiab] OR "pulmonary incompetence"[tiab] OR "tricuspid valve disease"[tiab] OR "tricuspid valve regurgitation"[tiab] OR "tricuspid valve insufficiency"[tiab] OR "tricuspid valve incompetence"[tiab] OR "tricuspid regurgitation"[tiab] OR "tricuspid insufficiency"[tiab] OR "tricuspid incompetence"[tiab] OR "tricuspid valve stenos*"[tiab] OR "tricuspid stenos*"[tiab])) OR "bicuspid aortic valve*"[tiab] OR "quadricuspid aortic valve"[tiab] OR "pulmonary atresia*"[tiab] OR "pulmonary valve atresia*"[tiab] OR "absent pulmonary valve"[tiab] OR "aortic atresia"[tiab] OR "absent aortic valve"[tiab] OR "tricuspid atresia*"[tiab] OR "tricuspid valve atresia*"[tiab] OR "absent right atrioventricular connection"[tiab] OR "pulmonary venous stenosis"[tiab] OR "double inlet left ventricle"[tiab] OR "mitral atresia"[tiab] OR "pulmonary artery stenosis"[tiab] OR "Shone* complex*"[tiab]) AND ("Neurophysiological Monitoring"[Mesh] OR "neuromonitoring"[tiab] OR "neurophysiological monitoring"[tiab] OR "neurophysiologic monitoring"[tiab] OR "cerebral monitoring"[tiab] OR "neurological monitoring"[tiab] OR "brain monitoring"[tiab] OR "neurologic monitoring"[tiab] OR "Electroencephalography"[Mesh:NoExp] OR "electroencephalogr*"[tiab] OR "electro encephalogr*"[tiab] OR "EEG"[tiab] OR "aEEG"[tiab] OR "cEEG"[tiab] OR "Spectroscopy, Near-Infrared"[Mesh] OR "NIRS"[tiab] OR "near infrared spectro*"[tiab] OR "NIR spectro*"[tiab] OR "Cell-Free Nucleic Acids"[Mesh] OR "cell-free DNA" [tiab] OR "circulating DNA"[tiab] OR "cfDNA"[tiab] OR "ccfDNA"[tiab] OR "DNA methylation patterns"[tiab] OR "brain-specific DNA"[tiab] OR "Biomarkers"[Mesh:NoExp] OR "Genetic Markers"[Mesh] OR "genetic marker*"[tiab] OR "biomarker*"[tiab] OR "biological marker*"[tiab] OR "biologic marker*"[tiab] OR "serum marker*"[tiab] OR "laboratory marker*"[tiab] OR "s100B"[tiab] OR "s100β"[tiab] OR "Glial ﬁbrillary acidic protein"[tiab] OR "GFAP "[tiab] OR "NSE"[tiab] OR "Neuron specific enolase"[tiab] OR "Brain derived neurotrophic factor"[tiab] OR "BDNF"[tiab]) NOT "Case Reports"[pt]

- 1. ***Embase***

("Heart Defects, Congenital"/exp OR 'congenital heart disease*':ti,ab,kw OR 'congenital cardi*':ti,ab,kw OR 'congenital heart defect*':ti,ab,kw OR 'congenital heart disorder*':ti,ab,kw OR 'heart malformation*':ti,ab,kw OR 'malformation* of heart*':ti,ab,kw OR 'malformation* of the heart':ti,ab,kw OR 'cyanotic heart disease':ti,ab,kw OR 'cyanotic heart disorder*':ti,ab,kw OR 'cyanotic heart defect*':ti,ab,kw OR 'cyanotic cardi*':ti,ab,kw OR 'pediatric heart disease*':ti,ab,kw OR 'paediatric heart disease*':ti,ab,kw OR 'paediatric cardi*':ti,ab,kw OR 'pediatric cardi*':ti,ab,kw OR "Aortic coarctation"/exp OR 'aortic coarctation':ti,ab,kw OR 'coarctation of the aorta':ti,ab,kw OR 'aorta coarctation*':ti,ab,kw OR 'hypoplastic aortic arch':ti,ab,kw OR 'interrupted aortic arch':ti,ab,kw OR 'aortic arch interruption':ti,ab,kw OR 'coarctatio aortae':ti,ab,kw OR

'aortico ventricular tunnel':ti,ab,kw OR 'aortico ventricular tunnels':ti,ab,kw OR 'aorticoventricular tunnel*':ti,ab,kw OR 'aortoventricular tunnel*':ti,ab,kw OR 'aortic-ventricular tunnel*':ti,ab,kw OR 'aorto ventricular tunnel*':ti,ab,kw OR 'cor triatriatum':ti,ab,kw OR 'subdivided left atrium':ti,ab,kw OR 'triatrial heart*':ti,ab,kw OR ('congenital':ti,ab,kw AND ("coronary vessel malformation"/exp OR 'coronary vessel anomal*':ti,ab,kw OR 'coronary vessel malformation':ti,ab,kw)) OR "situs inversus"/exp OR 'situs inversus':ti,ab,kw OR 'dextrocardia':ti,ab,kw OR 'Kartagener':ti,ab,kw OR 'levocardia':ti,ab,kw OR "ciliary dyskinesia"/exp OR 'ciliary diskinesia':ti,ab,kw OR 'patent ductus arteriosus':ti,ab,kw OR 'patency of the ductus arteriosus':ti,ab,kw OR 'patent ductus botalli':ti,ab,kw OR 'open ductus botalli':ti,ab,kw OR 'ductus arteriosus persisten*':ti,ab,kw OR 'persistent ductus arteriosus ':ti,ab,kw OR 'persistent ductus botalli':ti,ab,kw OR 'ductus arteriosus patency':ti,ab,kw OR 'ductus botalli patency':ti,ab,kw OR 'Patent foramen ovale':ti,ab,kw OR 'crisscross heart*':ti,ab,kw OR 'criss cross heart*':ti,ab,kw OR 'Ebstein*':ti,ab,kw OR 'ectopia cordis':ti,ab,kw OR 'ectocardia':ti,ab,kw OR 'ectopic heart':ti,ab,kw OR 'exocardia:ti,ab,kw' OR 'cardiac ectop*':ti,ab,kw OR 'Eisenmenger':ti,ab,kw OR 'Heart septal defect*':ti,ab,kw OR 'Heart septum defect*':ti,ab,kw OR 'cardiac septum defect*':ti,ab,kw OR 'cardiac septal defect*':ti,ab,kw OR 'Aortopulmonary Septal Defect*':ti,ab,kw OR 'Aortopulmonary septum Defect*':ti,ab,kw OR 'aorticopulmonary septum Defect*':ti,ab,kw OR 'aorticopulmonary Septal Defect*':ti,ab,kw OR 'truncus arteriosus':ti,ab,kw OR 'endocardial cushion defect*':ti,ab,kw OR 'atrioventricular canal':ti,ab,kw OR 'atrial septal defect*':ti,ab,kw OR 'atrial septum defect*':ti,ab,kw OR 'persistent ostium primum':ti,ab,kw OR 'patent foramen ovale':ti,ab,kw OR 'patent oval foramen':ti,ab,kw OR 'lutembacher':ti,ab,kw OR 'ventricular septal defect*':ti,ab,kw OR 'ventricular septum defect*':ti,ab,kw OR 'intraventricular septal defect*':ti,ab,kw OR 'intraventricular septum defect*':ti,ab,kw OR 'double outlet right ventricle':ti,ab,kw OR 'Taussig-Bing Anomaly':ti,ab,kw OR 'atrioventricular septal defect*':ti,ab,kw OR 'atrioventricular septum defect*':ti,ab,kw OR 'atrio ventricular septal defect*':ti,ab,kw OR 'atrio ventricular septum defect*':ti,ab,kw OR "Heterotaxy syndrome"/exp OR 'heterotaxy syndrome':ti,ab,kw OR 'visceral heterotax*':ti,ab,kw OR 'situus ambiguous viscerum':ti,ab,kw OR 'atrial isomerism*':ti,ab,kw OR 'Ivemark syndrome':ti,ab,kw OR 'HLHS':ti,ab,kw OR 'hypoplastic left heart':ti,ab,kw OR 'hypoplastic right heart':ti,ab,kw OR 'single ventricle':ti,ab,kw OR 'univentricular heart':ti,ab,kw OR 'monoventricular heart':ti,ab,kw OR 'non compaction of the ventricular myocardium':ti,ab,kw OR 'noncompaction of the ventricular myocardium':ti,ab,kw OR 'Fallots':ti,ab,kw OR 'Fallot':ti,ab,kw OR 'transposition of the great arter*':ti,ab,kw OR 'transposition of the great vessels':ti,ab,kw OR 'great vessels transposition*':ti,ab,kw OR 'great arter* transposition*':ti,ab,kw OR 'transposition of great arter*':ti,ab,kw OR 'transposition of great vessels':ti,ab,kw OR 'levotransposition*':ti,ab,kw OR 'dextrotransposition*':ti,ab,kw OR (('congenital':ti,ab,kw OR 'pediatric':ti,ab,kw OR 'paediatric':ti,ab,kw) AND ("cardiomyopathy"/de OR 'cardiomyopathy':ti,ab,kw OR 'cardiomyopathies':ti,ab,kw)) OR 'arrhythmogenic right ventricular dysplasia':ti,ab,kw OR 'arrhythmogenic right ventricular cardiomyopath*':ti,ab,kw OR "Scimitar syndrome"/exp OR 'anomalous pulmonary venous return':ti,ab,kw OR 'scimitar':ti,ab,kw OR 'tapvr':ti,ab,kw OR 'papvr':ti,ab,kw OR "persistent left superior vena cava"/exp OR 'left superior vena cava':ti,ab,kw OR 'left sided superior vena cava':ti,ab,kw OR 'bilateral superior vena cava':ti,ab,kw OR 'superior vena cava duplication':ti,ab,kw OR 'persistent superior vena cava':ti,ab,kw OR 'bilateral superior caval veins':ti,ab,kw OR 'bilateral SVC':ti,ab,kw OR 'SVC duplication':ti,ab,kw OR "vascular ring"/exp OR 'vascular ring*':ti,ab,kw OR 'pulmonary artery sling':ti,ab,kw OR 'double aortic arch':ti,ab,kw OR 'right aortic arch':ti,ab,kw OR (('congenital':ti,ab,kw OR 'pediatric':ti,ab,kw OR 'paediatric':ti,ab,kw) AND ('Valvular heart disease"/exp OR 'heart valve disease*':ti,ab,kw OR 'heart valvular disease*':ti,ab,kw OR 'valvular heart disease*':ti,ab,kw OR 'aortic valve disease*':ti,ab,kw OR "aortic valve disease"/exp OR 'aortic heart disease*':ti,ab,kw OR 'aortic valvular heart disease*':ti,ab,kw OR 'aortic valve insufficiency':ti,ab,kw OR 'aortic valve incompetence':ti,ab,kw OR 'aortic regurgitation':ti,ab,kw OR 'aortic valve regurgitation':ti,ab,kw OR 'aortic incompetence':ti,ab,kw OR 'aortic insufficiency':ti,ab,kw OR 'aortic valve prolapse*':ti,ab,kw OR 'aortic valve stenos*':ti,ab,kw OR 'aortic stenos*':ti,ab,kw OR 'mitral valve prolapse*':ti,ab,kw OR 'tricuspid valve prolapse':ti,ab,kw OR 'mitral valve disease':ti,ab,kw OR 'mitral valve insufficiency':ti,ab,kw OR 'mitral valve incompetence':ti,ab,kw OR 'mitral regurgitation':ti,ab,kw OR 'mitral valve regurgitation':ti,ab,kw OR 'mitral incompetence':ti,ab,kw OR 'mitral insufficiency':ti,ab,kw OR 'mitral valve stenos*':ti,ab,kw OR 'mitral stenos*':ti,ab,kw OR 'pulmonary valve stenos*':ti,ab,kw OR 'pulmonary stenos*':ti,ab,kw OR 'pulmonic stenos*':ti,ab,kw OR 'pulmonary valve disease':ti,ab,kw OR 'pulmonary valve regurgitation':ti,ab,kw OR 'pulmonary valve insufficiency':ti,ab,kw OR 'pulmonary valve incompetence':ti,ab,kw OR 'pulmonary regurgitation':ti,ab,kw OR 'pulmonary insufficiency':ti,ab,kw OR 'pulmonary incompetence':ti,ab,kw OR 'tricuspid valve disease':ti,ab,kw OR 'tricuspid valve regurgitation':ti,ab,kw OR 'tricuspid valve insufficiency':ti,ab,kw OR 'tricuspid valve incompetence':ti,ab,kw OR 'tricuspid regurgitation':ti,ab,kw OR 'tricuspid insufficiency':ti,ab,kw OR 'tricuspid incompetence':ti,ab,kw OR 'tricuspid valve stenos*':ti,ab,kw OR 'tricuspid stenos*':ti,ab,kw)) OR 'quadricuspid aortic valve':ti,ab,kw OR 'bicuspid aortic valve':ti,ab,kw OR 'pulmonary atresia*':ti,ab,kw OR 'pulmonary valve atresia*':ti,ab,kw OR 'absent pulmonary valve':ti,ab,kw OR 'aortic atresia':ti,ab,kw OR 'absent aortic valve':ti,ab,kw OR 'tricuspid atresia*':ti,ab,kw OR 'tricuspid valve atresia*':ti,ab,kw OR 'absent right atrioventricular connection':ti,ab,kw OR 'pulmonary venous stenosis':ti,ab,kw OR 'double inlet left ventricle':ti,ab,kw OR 'mitral atresia':ti,ab,kw OR 'pulmonary artery stenosis':ti,ab,kw OR 'Shone* complex*':ti,ab,kw) AND ("Neuromonitoring"/exp OR "Neurophysiological monitoring"/exp OR 'neuromonitoring':ti,ab,kw OR 'neurophysiological monitoring':ti,ab,kw OR 'neurophysiologic monitoring':ti,ab,kw OR 'cerebral monitoring':ti,ab,kw OR 'neurological monitoring':ti,ab,kw OR 'brain monitoring':ti,ab,kw OR 'neurologic monitoring':ti,ab,kw OR "Electroencephalography"/de OR "Continuous Electroencephalography"/exp OR 'electroencephalogr*':ti,ab,kw OR 'electro encephalogr*':ti,ab,kw OR 'EEG':ti,ab,kw OR 'aEEG':ti,ab,kw OR 'cEEG':ti,ab,kw OR "Near Infrared Spectroscopy"/exp OR 'NIRS':ti,ab,kw OR 'near infrared spectro*':ti,ab,kw OR 'NIR spectro*':ti,ab,kw OR "Cell Free Nucleic Acid"/de OR "Circulating free DNA"/exp OR 'cell-free DNA':ti,ab,kw OR 'circulating DNA':ti,ab,kw OR 'cfDNA':ti,ab,kw OR 'ccfDNA':ti,ab,kw OR 'DNA methylation patterns':ti,ab,kw OR 'brain-specific DNA':ti,ab,kw OR "Biological Marker"/exp OR "Genetic marker"/de OR "Marker Gene"/exp OR "DNA marker"/exp OR 'genetic marker*':ti,ab,kw OR 'biomarker*':ti,ab,kw OR 'biological marker*':ti,ab,kw OR 'biologic marker*':ti,ab,kw OR 'serum marker*':ti,ab,kw OR 'laboratory marker*':ti,ab,kw OR 's100B':ti,ab,kw OR 's100β':ti,ab,kw OR 'Glial ﬁbrillary acidic protein':ti,ab,kw OR 'GFAP':ti,ab,kw OR 'NSE':ti,ab,kw OR 'Neuron specific enolase':ti,ab,kw OR 'Brain derived neurotrophic factor':ti,ab,kw OR 'BDNF':ti,ab,kw) NOT ('conference abstract':it OR 'case report':it)

- 1. ***CENTRAL***

([mh "Heart Defects, Congenital"] OR [mh "Situs Inversus"] OR [mh "Scimitar Syndrome"] OR [mh "Persistent Left Superior Vena Cava"] OR [mh "Vascular Ring"] OR ((congenital OR pediatric OR paediatric) NEAR/1 ([mh "Heart Valve Diseases"] OR [mh "Cardiomyopathies"])) OR ((congenital NEXT heart NEXT defect*) OR (congenital NEXT heart NEXT disease*) OR (congenital NEXT heart NEXT disorder*) OR (congenital NEXT cardi*) OR (heart NEXT abnormalit*) OR (heart NEXT malformation*) OR (malformation NEXT of NEXT heart*) OR "malformation of the heart" OR (cyanotic NEXT heart NEXT disease*) OR (cyanotic NEXT heart NEXT defect*) OR (cyanotic NEXT heart NEXT disorder*) OR (cyanotic NEXT cardi*) OR (pediatric NEXT heart NEXT disease*) OR (paediatric NEXT heart NEXT disease) OR (pediatric NEXT cardi*) OR (paediatric NEXT cardi*) OR "aortic coarctation" OR "coarctation of the aorta" OR "aorta coarctation" OR "hypoplastic aortic arch" OR "interrupted aortic arch" OR "aortic arch interruption" OR "coarctatio aortae" OR (aortico NEAR/1 "ventricular tunnel") OR (aortico NEAR/1 "ventricular tunnels") OR (aorticoventricular NEXT tunnel*) OR (aortoventricular NEXT tunnel*) OR (aortic NEXT ventricular NEXT tunnel*) OR (aorto NEXT ventricular NEXT tunnel*) OR "cor triatriatum" OR "subdivided left atrium" OR (triatrial NEXT heart*) OR (congenital NEXT ((coronary NEXT vessel NEXT anomal*) OR "coronary vessel malformation")) OR (crisscross NEXT heart*) OR (criss NEXT cross NEXT heart*) OR "situs inversus" OR "dextrocardia" OR "Kartagener" OR "ciliary dyskinesia" OR "levocardia" OR "patent ductus arteriosus" OR "patency of the ductus arteriosus" OR "patent ductus botalli" OR "open ductus botalli" OR (ductus NEXT arteriosus NEXT persisten*) OR "persistent ductus arteriosus " OR "persistent ductus botalli" OR "ductus arteriosus patency" OR "ductus botalli patency" OR "Patent foramen ovale" OR Ebstein* OR "Ectopia cordis" OR Ectocardia OR "ectopic heart" OR exocardia OR (cardiac NEXT ectop*) OR Eisenmenger* OR (Heart NEXT septal NEXT defect*) OR (Heart NEXT septum NEXT defect*) OR (cardiac NEXT septum NEXT defect*) OR (cardiac NEXT septal NEXT defect*) OR (Aortopulmonary NEXT Septal NEXT Defect*) OR (Aortopulmonary NEXT septum NEXT Defect*) OR (aorticopulmonary NEXT septum NEXT Defect*) OR (aorticopulmonary NEXT Septal NEXT Defect*) OR "truncus arteriosus" OR (endocardial NEXT cushion NEXT defect*) OR "atrioventricular canal" OR (atrial NEXT septal NEXT defect*) OR (atrial NEXT septum NEXT defect*) OR "persistent ostium primum" OR (ostium NEXT secundum*) OR "patent foramen ovale" OR "patent oval foramen" OR "lutembacher" OR (ventricular NEXT septal NEXT defect*) OR (ventricular NEXT septum NEXT defect*) OR (intraventricular NEXT septal NEXT defect*) OR (intraventricular NEXT septum NEXT defect*) OR "double outlet right ventricle" OR "Taussig Bing Anomaly" OR (atrioventricular NEXT septal NEXT defect*) OR (atrioventricular NEXT septum NEXT defect*) OR (heterotaxy NEXT syndrome*) OR (visceral NEXT heterotax*) OR "situs ambiguus viscerum" OR (atrial NEXT isomerism*) OR "Ivemark Syndrome" OR "HLHS" OR "hypoplastic left heart" OR "univentricular heart" OR "hypoplastic right heart" OR "single ventricle" OR "univentricular heart" OR "monoventricular heart" OR "non compaction of the ventricular myocardium" OR "noncompaction of the ventricular myocardium" OR Fallot OR Fallots OR (transposition NEXT of NEXT the NEXT great NEXT arter*) OR "transposition of the great vessels" OR (great NEXT vessels NEXT transposition*) OR (great NEXT arter* NEXT transposition*) OR "TGA" OR (transposition NEXT of NEXT great NEXT arter*) OR "transposition of great vessels" OR levotransposition* OR dextrotransposition* OR ((congenital OR pediatric OR paediatric) NEAR/1 cardiomyopath*) OR (arrhythmogenic NEXT right NEXT ventricular NEXT cardiomyopath*) OR "arrhythmogenic right ventricular dysplasia" OR "anomalous pulmonary venous return" OR Scimitar OR TAPVR OR "pulmonary venous return anomaly" OR "PAPVR" OR "left superior vena cava" OR "left sided superior vena cava" OR "persistent superior vena cava" OR "Bilateral superior vena cava" OR "Bilateral superior caval veins" OR "superior vena cava duplication" OR "bilateral SVC" OR (SVC NEXT duplication*) OR (vascular NEXT ring*) OR "pulmonary artery sling" OR "double aortic arch" OR "right aortic arch" OR ((congenital OR pediatric OR paediatric) AND ((heart NEXT valve NEXT disease*) OR (heart NEXT valvular NEXT disease*) OR (valvular NEXT heart NEXT disease*) OR (aortic NEXT valve NEXT disease*) OR (aortic NEXT heart NEXT disease*) OR (aortic NEXT valvular NEXT heart NEXT disease*) OR "aortic valve insufficiency" OR "aortic valve incompetence" OR "aortic regurgitation" OR "aortic valve regurgitation" OR "aortic incompetence" OR "aortic insufficiency" OR (aortic NEXT valve NEXT prolapse*) OR (aortic NEXT valve NEXT stenos*) OR (aortic NEXT stenos*) OR (mitral NEXT valve NEXT prolapse*) OR "tricuspid valve prolapse" OR "mitral valve disease" OR "mitral valve insufficiency" OR "mitral valve incompetence" OR "mitral regurgitation" OR "mitral valve regurgitation" OR "mitral incompetence" OR "mitral insufficiency" OR (mitral NEXT valve NEXT stenos*) OR (mitral NEXT stenos*) OR (pulmonary NEXT valve NEXT stenos*) OR (pulmonary NEXT stenos*) OR (pulmonic NEXT stenos*) OR "pulmonary valve disease" OR "pulmonary valve regurgitation" OR "pulmonary valve insufficiency" OR "pulmonary valve incompetence" OR "pulmonary regurgitation" OR "pulmonary insufficiency" OR "pulmonary incompetence" OR "tricuspid valve disease" OR "tricuspid valve regurgitation" OR "tricuspid valve insufficiency" OR "tricuspid valve incompetence" OR "tricuspid regurgitation" OR "tricuspid insufficiency" OR "tricuspid incompetence" OR (tricuspid NEXT valve NEXT stenos*) OR (tricuspid NEXT stenos*))) OR (bicuspid NEXT aortic NEXT valve*) OR "quadricuspid aortic valve" OR (pulmonary NEXT atresia*) OR (pulmonary NEXT valve NEXT atresia*) OR "absent pulmonary valve" OR "aortic atresia" OR "absent aortic valve" OR (tricuspid NEXT atresia*) OR (tricuspid NEXT valve NEXT atresia*) OR "absent right atrioventricular connection" OR "pulmonary venous stenosis" OR "double inlet left ventricle" OR "mitral atresia" OR "pulmonary artery stenosis" OR "Shone complex"):ti,ab,kw) AND ([mh "Neurophysiological Monitoring"] OR [mh ^"Electroencephalography"] OR [mh "Spectroscopy, Near-Infrared"] OR [mh "Cell-Free Nucleic Acids"] OR [mh ^"Biomarkers"] OR [mh "Cellular markers"] OR (neuromonitoring OR "neurophysiological monitoring" OR "neurophysiologic monitoring" OR "cerebral monitoring" OR "neurological monitoring" OR "brain monitoring" OR (electro NEXT encephalogr*) OR electroencephalogr* OR EEG OR aEEG OR cEEG OR NIRS OR "near infrared spectroscopy" OR "near infrared spectrometry" OR (NIR NEXT spectro*) OR "cell free DNA" OR "circulating DNA" OR cfDNA OR ccfDNA OR "DNA methylation patterns" OR "brain specific DNA" OR Biomarker* OR (genetic NEXT marker*) OR (biological NEXT marker*) OR (biologic NEXT marker*) OR (serum NEXT marker*) OR (laboratory NEXT marker*) OR s100B OR "s100β" OR "Glial ﬁbrillary acidic protein" OR "GFAP " OR "NSE" OR "Neuron specific enolase" OR "Brain derived neurotrophic factor" OR "BDNF"):ti,ab,kw)

- 1. ***Web of Science***

(TS=(“congenital heart defect*” OR “congenital heart disease*” OR “congenital cardiac disease*” OR “congenital heart disorder*” OR “congenital cardi*” OR “heart abnormalit*” OR “heart malformation*” OR “malformation* of heart*” OR “malformation* of the heart” OR “cyanotic heart disease*” OR “cyanotic heart defect*” OR “cyanotic heart disorder*” OR “cyanotic cardi*” OR “pediatric heart disease” OR “paediatric heart disease” OR “paediatric cardi*” OR “pediatric cardi*” OR "aortic coarctation" OR "coarctation of the aorta" OR "aorta coarctation" OR "hypoplastic aortic arch" OR "interrupted aortic arch" OR “aortic arch interruption” OR “coarctatio aortae" OR (“aortico ventricular” NEAR/1 “tunnel*”) OR (“aorticoventricular” NEAR/1 “tunnel*”) OR (“aortoventricular” NEAR/1 “tunnel*”) OR “aortic-ventricular tunnel*” OR “aorto-ventricular tunnel*” OR “cor triatriatum” OR “subdivided left atrium” OR “triatrial heart*” OR ((congenital) AND (“coronary vessel anomal*” OR “coronary NEAR/1 malformation”)) OR “crisscross heart*” OR “criss-cross heart*” OR “situs inversus” OR “dextrocardia” OR “Kartagener” OR “ciliary dyskinesia” OR “levocardia” OR "patent ductus arteriosus" OR "patency of the ductus arteriosus" OR “patent ductus botalli" OR “open ductus botalli" OR “ductus arteriosus persisten*” OR “persistent ductus arteriosus" OR “persistent ductus botalli" OR “ductus arteriosus patency" OR “ductus botalli patency" OR “patent foramen ovale” OR “Ebstein*” OR “Ectopia cordis” OR “Ectocardia” OR “ectopic heart” OR “exocardia” OR “cardiac ectop*” OR “Eisenmenger*” OR “Heart septal defect*” OR “Heart septum defect*” OR “cardiac septum defect*” OR “cardiac septal defect*” OR “Aortopulmonary Septal Defect*” OR “aortopulmonary septum defect*” OR “aorticopulmonary Septal Defect*” OR “aorticopulmonary septum Defect*” OR “truncus arteriosus” OR “endocardial cushion defect*” OR “atrioventricular canal” OR “atrial septal defect*” OR “atrial septum defect*” OR “persistent ostium primum” OR “ostium secundum atrial septal defect*” OR “patent foramen ovale” OR “patent oval foramen” OR “lutembacher” OR “ventricular septal defect*” OR “ventricular septum defect*” OR “intraventricular septal defect*” OR “intraventricular septum defect*” OR “double-outlet right ventricle” OR “Taussig-Bing Anomaly” OR “atrioventricular septal defect*” OR “atrioventricular septum defect*” OR “heterotaxy syndrome*” OR “visceral heterotax*” OR “situs ambiguus viscerum” OR “atrial isomerism*” OR “Ivemark Syndrome” OR “HLHS” OR “hypoplastic left heart” OR “univentricular heart” OR “hypoplastic right heart” OR “single ventricle” OR “univentricular heart” OR “monoventricular heart” OR “non-compaction of the ventricular myocardium” OR “noncompaction of the ventricular myocardium” OR “Fallot” OR “Fallots” OR “transposition of the great arter*” OR “transposition of the great vessels” OR “great vessels transposition*” OR “great artery* transposition*” OR “TGA” OR “transposition of great arter*” OR “transposition of great vessels” OR “levotransposition*” OR “dextrotransposition*” OR “congenital cardiomyopath*” OR “pediatric cardiomyopath*” OR “paediatric cardiomyopath*” OR ((‘congenital’ OR “pediatric” OR “paediatric”) NEAR/1 (cardiomyopath*)) OR “arrhythmogenic right ventricular cardiomyopath*” OR “arrhythmogenic right ventricular dysplasia” OR “anomalous pulmonary venous return” OR “Scimitar” OR “TAPVR” OR “pulmonary venous return anomaly” OR “PAPVR” OR “left superior vena cava” OR “left sided superior vena cava” OR “Bilateral superior vena cava” OR “Bilateral superior caval veins” OR “superior vena cava duplication” OR “bilateral SVC” OR “SVC duplication*” OR “persistent superior vena cava” OR “vascular ring*” OR “pulmonary artery sling” OR “double aortic arch” OR “right aortic arch” OR ((“congenital” OR “pediatric” OR “paediatric”) AND (“heart valve disease*” OR “heart valvular disease*” OR “valvular heart disease*” OR “aortic valve disease*” OR “aortic heart disease*” OR “aortic valvular heart disease*” OR “aortic valve insufficiency” OR “aortic valve incompetence” OR “aortic regurgitation” OR “aortic valve regurgitation” OR “aortic incompetence” OR “aortic insufficiency” OR “aortic valve prolapse*” OR “aortic valve stenos*” OR “aortic stenos*” OR “mitral valve prolapse*” OR “tricuspid valve prolapse” OR “mitral valve disease” OR “mitral valve insufficiency” OR “mitral valve incompetence” OR “mitral regurgitation” OR “mitral valve regurgitation” OR “mitral incompetence” OR “mitral insufficiency” OR “mitral valve stenos*” OR “mitral stenos*” OR “pulmonary valve stenos*” OR “pulmonary stenos*” OR “pulmonic stenos*” OR “pulmonary valve disease” OR “pulmonary valve regurgitation” OR “pulmonary valve insufficiency” OR “pulmonary valve incompetence” OR “pulmonary regurgitation” OR “pulmonary insufficiency” OR “pulmonary incompetence” OR “tricuspid valve disease” OR “tricuspid valve regurgitation” OR “tricuspid valve insufficiency” OR “tricuspid valve incompetence” OR “tricuspid regurgitation” OR “tricuspid insufficiency” OR “tricuspid incompetence” OR “tricuspid valve stenos*” OR “tricuspid stenos*”)) OR “bicuspid aortic valve*” OR “quadricuspid aortic valve” OR “pulmonary atresia*” OR “pulmonary valve atresia*” OR “absent pulmonary valve” OR “aortic atresia” OR “absent aortic valve” OR “tricuspid atresia*” OR “tricuspid valve atresia*” OR “absent right atrioventricular connection” OR “pulmonary venous stenosis” OR “double inlet left ventricle” OR “mitral atresia” OR “pulmonary artery stenosis” OR “Shone* complex*”)) AND TS=(“Neurophysiological Monitoring” OR “neuromonitoring” OR “neurophysiological monitoring” OR “neurophysiologic monitoring” OR “cerebral monitoring” OR “neurological monitoring” OR “neurologic monitoring” OR “brain monitoring” OR “electroencephalogr*” OR “electro encephalogr*” OR “EEG” OR “aEEG” OR “cEEG” OR “Spectroscopy, Near-Infrared” OR “NIRS” OR “near infrared spectro*” OR “NIR spectro*” OR “Cell-Free Nucleic Acids” OR “cell-free DNA” OR “circulating DNA” OR “cfDNA” OR “ccfDNA” OR “DNA methylation patterns” OR “brain-specific DNA” OR “Biomarker*” OR “genetic marker*” OR “biological marker*” OR “biologic marker*” OR “serum marker*” OR “laboratory marker*” OR “s100B” OR “s100β” OR “Glial ﬁbrillary acidic protein” OR “GFAP” OR “NSE” OR “Neuron specific enolase” OR “Brain-derived neurotrophic factor” OR “BDNF”)) NOT DT=(“meeting abstract”)

- 1. ***Clinicaltrials.gov***

("congenital heart defect" OR "congenital heart defects" OR "congenital heart disease" OR "congenital heart diseases" OR "congenital cardiopathy" OR "congenital cardiopathies" OR "congenital heart disorder" OR "congenital heart disorders" OR "heart abnormality" OR "heart abnormalities" OR "heart malformation"OR "heart malformations" OR "malformation of heart" OR "malformation of the heart" OR "malformations of the heart" OR "cyanotic heart disease" OR "cyanotic heart diseases" OR "cyanotic cardiopathy" OR "cyanotic cardiopathies" OR "cyanotic heart defect" OR "cyanotic heart defects" OR "cyanotic heart disorder" OR "cyanotic heart disorders" OR "pediatric heart disease" OR "pediatric heart diseases" OR "paediatric heart disease" OR "paediatric heart diseases" OR "pediatric cardiopathy" OR "pediatric cardiopathies" OR "aortic coarctation" OR "coarctation of the aorta" OR "aorta coarctation" OR "hypoplastic aortic arch" OR "interrupted aortic arch" OR "aortic arch interruption" OR "coarctatio aortae" OR "aorticoventricular tunnel" OR "aorticoventricular tunnels" OR "aortoventricular tunnel" OR "aortoventricular tunnels" OR "aortic ventricular tunnel" OR "aortic ventricular tunnel" OR "cor triatriatum" OR "subdivided left atrium" OR "triatrial heart" OR (congenital AND ("coronary vessel anomaly" OR "coronary vessel anomalies" OR "coronary vessel malformation" OR "coronary vessel malformations")) OR "crisscross heart" OR "Situs Inversus" OR "dextrocardia" OR "Kartagener" OR "ciliary dyskinesia" OR "levocardia" OR "patent ductus arteriosus" OR "patency of the ductus arteriosus" OR "patent ductus botalli" OR "open ductus botalli" OR "ductus arteriosus persistens" OR "ductus arteriosus persistence" OR "persistent ductus arteriosus" OR "persistent ductus botalli" OR "ductus arteriosus patency" OR "ductus botalli patency" OR "patent foramen ovale" OR "Ebstein" OR "Ectopia cordis" OR "ectocardia" OR "ectopic heart" OR "exocardia" OR "cardiac ectopy" OR "Eisenmenger" OR "Heart septal defect" OR "Heart septum defect" OR "cardiac septum defect" OR "cardiac septal defect" OR "Aortopulmonary Septal Defect" OR "Aortopulmonary septum Defect*"OR "aorticopulmonary Septal Defect*"OR "aorticopulmonary septum defect" OR "truncus arteriosus" OR "endocardial cushion defect" OR "atrioventricular canal" OR "atrial septal defect" OR "atrial septum defect" OR "persistent ostium primum" OR "patent foramen ovale" OR "patent oval foramen" OR "lutembacher" OR "ventricular septal defect" OR "ventricular septum defect" OR "intraventricular septal defect" OR "intraventricular septum defect" OR "double outlet right ventricle" OR "Taussig-Bing Anomaly" OR "atrioventricular septal defect" OR "atrioventricular septum defect" OR "heterotaxy syndrome" OR "visceral heterotaxy" OR "situs ambiguus viscerum" OR "atrial isomerism" OR "Ivemark Syndrome" OR "HLHS" OR "hypoplastic left heart" OR "hypoplastic right heart" OR "single ventricle" OR "univentricular heart" OR "monoventricular heart" OR "non compaction ventricular myocardium" OR "noncompaction ventricular myocardium" OR "Fallots" OR "Fallot" OR "transposition of the great arteries" OR "transposition of the great vessels" OR "great vessels transposition" OR "great arteries transposition" OR "transposition of great arteries" OR "transposition of great vessels" OR "levotransposition" OR "dextrotransposition" OR (("congenital" OR "pediatric" OR "paediatric") AND "Cardiomyopathies") OR "congenital cardiomyopathy" OR "congenital cardiomyopathies" OR "pediatric cardiomyopathy" OR "paediatric cardiomyopathies" OR "paediatric cardiomyopathy" OR "pediatric cardiomyopathies" OR "arrhythmogenic right ventricular cardiomyopathy" OR "arrhythmogenic right ventricular dysplasia" OR "Scimitar Syndrome" OR "anomalous pulmonary venous return" OR "Scimitar" OR "TAPVR" OR "pulmonary venous return anomaly"OR "pulmonary venous return anomalies" OR "PAPVR" OR "Persistent Left Superior Vena Cava" OR "left superior vena cava" OR "left-sided superior vena cava" OR "Bilateral superior vena cava" OR "Bilateral superior caval veins" OR "superior vena cava duplication" OR "bilateral SVC" OR "SVC duplication" OR "persistent superior vena cava" OR "Vascular Ring" OR "vascular ring" OR "pulmonary artery sling" OR "double aortic arch" OR "right aortic arch" OR (("congenital" OR "pediatric" OR "paediatric") AND ("heart valve disease" OR "heart valvular disease" OR "valvular heart disease" OR "aortic valve disease" OR "aortic heart disease" OR "aortic valvular heart disease" OR "aortic valve insufficiency" OR "aortic valve incompetence" OR "aortic regurgitation" OR "aortic valve regurgitation" OR "aortic incompetence" OR "aortic insufficiency" OR "aortic valve prolapse" OR "aortic valve stenosis" OR "aortic stenosis" OR "mitral valve prolapse" OR "tricuspid valve prolapse" OR "mitral valve disease" OR "mitral valve insufficiency" OR "mitral valve incompetence" OR "mitral regurgitation" OR "mitral valve regurgitation" OR "mitral incompetence" OR "mitral insufficiency" OR "mitral valve stenosis" OR "mitral stenosis" OR "pulmonary valve stenosis" OR "pulmonary stenosis" OR "pulmonic stenosis" OR "pulmonary valve disease" OR "pulmonary valve regurgitation" OR "pulmonary valve insufficiency" OR "pulmonary valve incompetence" OR "pulmonary regurgitation" OR "pulmonary insufficiency" OR "pulmonary incompetence" OR "tricuspid valve disease" OR "tricuspid valve regurgitation" OR "tricuspid valve insufficiency"OR "tricuspid valve incompetence"OR "tricuspid regurgitation" OR "tricuspid insufficiency" OR "tricuspid incompetence" OR "tricuspid valve stenosis"OR "tricuspid stenosis")) OR "bicuspid aortic valve"OR "quadricuspid aortic valve" OR "pulmonary atresia" OR "pulmonary valve atresia" OR "absent pulmonary valve" OR "aortic atresia" OR "absent aortic valve" OR "tricuspid atresia" OR "tricuspid valve atresia" OR "absent right atrioventricular connection" OR "pulmonary venous stenosis" OR "double inlet left ventricle" OR "mitral atresia" OR "pulmonary artery stenosis" OR "Shone complex") AND ("neuromonitoring" OR "neurophysiological monitoring" OR "neurophysiologic monitoring" OR "cerebral monitoring" OR "neurological monitoring" OR "brain monitoring" OR "neurologic monitoring" OR "electroencephalography" OR "EEG" OR "aEEG" OR "cEEG" OR "NIRS" OR "near infrared spectroscopy" OR "near infrared spectrometry" OR "NIR spectroscopy" OR "NIR spectrometry" OR "Cell-Free Nucleic Acids" OR "cell-free DNA" OR "circulating DNA" OR "cfDNA" OR "ccfDNA" OR "DNA methylation patterns" OR "brain-specific DNA" OR "Genetic Markers" OR "biomarker" OR "biomarkers" OR "biological marker" OR "biologic marker" OR "serum marker" OR "laboratory marker" OR "biological markers" OR "biologic markers" OR "serum markers" OR "laboratory markers" OR "s100B" OR "s100β" OR "Glial ﬁbrillary acidic protein" OR "GFAP " OR "NSE" OR "Neuron specific enolase" OR "Brain derived neurotrophic factor" OR "BDNF")

- 1. ***ICTRP***

("congenital heart defect*" OR "congenital heart disease*" OR "congenital cardi*" OR "congenital heart disorder*" OR "heart abnormalit*" OR "heart malformation*" OR "malformation* of heart*" OR "malformation* of the heart" OR "cyanotic heart disease*" OR "cyanotic cardi*" OR "cyanotic heart defect*" OR "cyanotic heart disorder" OR "cyanotic heart disorders" OR "pediatric heart disease*" OR "paediatric heart disease*" OR "pediatric cardi*" OR "aortic coarctation" OR "coarctation of the aorta" OR "aorta coarctation" OR "hypoplastic aortic arch" OR "interrupted aortic arch" OR "aortic arch interruption" OR "coarctatio aortae" OR "aortico ventricular tunnel" OR "aortico ventricular tunnels" OR "aorticoventricular tunnel*" OR "aortoventricular tunnel*" OR "aortic ventricular tunnel*" OR "aorto ventricular tunnel*" OR "cor triatriatum" OR "subdivided left atrium" OR "triatrial heart*" OR ((congenital) AND ("coronary vessel anomal*" OR "coronary vessel malformation" OR "coronary vessel malformations")) OR "crisscross heart*" OR "criss cross heart*" OR "situs inversus" OR "dextrocardia" OR "Kartagener" OR "ciliary dyskinesia" OR "levocardia" OR "patent ductus arteriosus" OR "patency of the ductus arteriosus" OR "patent ductus botalli" OR "open ductus botalli" OR "ductus arteriosus persisten*" OR "persistent ductus arteriosus " OR "persistent ductus botalli" OR "ductus arteriosus patency" OR "ductus botalli patency" OR "patent foramen ovale" OR "Ebstein*" OR "Ectopia cordis" OR "ectocardia" OR "ectopic heart" OR "exocardia" OR "cardiac ectop*" OR "Eisenmenger*" OR "Heart septal defect*" OR "Heart septum defect*" OR "cardiac septum defect*" OR "cardiac septal defect*" OR "Aortopulmonary Septal Defect*" OR "Aortopulmonary septum Defect*" OR "aorticopulmonary Septal Defect*" OR "aorticopulmonary septum defect" OR "truncus arteriosus" OR "endocardial cushion defect*" OR "atrioventricular canal" OR "atrial septal defect*" OR "atrial septum defect*" OR "persistent ostium primum" OR "patent foramen ovale" OR "patent oval foramen" OR "lutembacher" OR "ventricular septal defect*" OR "ventricular septum defect*" OR "intraventricular septal defect*" OR "intraventricular septum defect*" OR "double outlet right ventricle" OR "Taussig-Bing Anomaly" OR "atrioventricular septal defect*" OR "atrioventricular septum defect*" OR "atrio ventricular septal defect*" OR "atrio ventricular septum defect" OR "atrio ventricular septum defects" OR "heterotaxy syndrome*" OR "visceral heterotax*" OR "situs ambiguus viscerum" OR "atrial isomerism*" OR "Ivemark Syndrome" OR "HLHS" OR "hypoplastic left heart" OR "hypoplastic right heart" OR "single ventricle" OR "univentricular heart" OR "monoventricular heart" OR "non compaction ventricular myocardium" OR "noncompaction ventricular myocardium" OR "Fallots" OR "Fallot" OR "transposition of the great arter*" OR "transposition of the great vessels" OR "great vessels transposition*" OR "great arteries transposition*" OR "transposition of great arteries"[tiab:~1] OR "transposition of great vessels" OR "levotransposition*" OR "dextrotransposition*" OR (("congenital" OR "pediatric" OR "paediatric") AND ("cardiomyopathy" OR Cardiomyopathies")) OR "arrhythmogenic right ventricular cardiomyopath*" OR "arrhythmogenic right ventricular dysplasia" OR "anomalous pulmonary venous return" OR "Scimitar" OR "TAPVR" OR "pulmonary venous return anomal*" OR "PAPVR" OR "left superior vena cava" OR "left-sided superior vena cava" OR "Bilateral superior vena cava" OR "Bilateral superior caval veins" OR "superior vena cava duplication" OR "bilateral SVC" OR "SVC duplication*" OR "persistent superior vena cava" OR "vascular ring*" OR "pulmonary artery sling" OR "double aortic arch" OR "right aortic arch" OR (("congenital" OR "pediatric" OR "paediatric") AND ("heart valve disease*" OR "heart valvular disease*" OR "valvular heart disease*" OR "aortic valve disease*" OR "aortic heart disease*" OR "aortic valvular heart disease*" OR "aortic valve insufficiency" OR "aortic valve incompetence" OR "aortic regurgitation" OR "aortic valve regurgitation" OR "aortic incompetence" OR "aortic insufficiency" OR "aortic valve prolapse*" OR "aortic valve stenos*" OR "aortic stenos*" OR "mitral valve prolapse*" OR "tricuspid valve prolapse" OR "mitral valve disease" OR "mitral valve insufficiency" OR "mitral valve incompetence" OR "mitral regurgitation" OR "mitral valve regurgitation" OR "mitral incompetence" OR "mitral insufficiency" OR "mitral valve stenos*" OR "mitral stenos*" OR "pulmonary valve stenos*" OR "pulmonary stenos*" OR "pulmonic stenos*" OR "pulmonary valve disease" OR "pulmonary valve regurgitation" OR "pulmonary valve insufficiency" OR "pulmonary valve incompetence" OR "pulmonary regurgitation" OR "pulmonary insufficiency" OR "pulmonary incompetence" OR "tricuspid valve disease" OR "tricuspid valve regurgitation" OR "tricuspid valve insufficiency" OR "tricuspid valve incompetence" OR "tricuspid regurgitation" OR "tricuspid insufficiency" OR "tricuspid incompetence" OR "tricuspid valve stenos*" OR "tricuspid stenos*")) OR "bicuspid aortic valve*" OR "quadricuspid aortic valve" OR "pulmonary atresia*" OR "pulmonary valve atresia*" OR "absent pulmonary valve" OR "aortic atresia" OR "absent aortic valve" OR "tricuspid atresia*" OR "tricuspid valve atresia*" OR "absent right atrioventricular connection" OR "pulmonary venous stenosis" OR "double inlet left ventricle" OR "mitral atresia" OR "pulmonary artery stenosis" OR "Shone* complex*") AND ("neuromonitoring" OR "neurophysiological monitoring" OR "neurophysiologic monitoring" OR "cerebral monitoring" OR "neurological monitoring" OR "brain monitoring" OR "neurologic monitoring" OR "electroencephalogr*" OR "electro encephalogr*" OR "EEG" OR "aEEG" OR "cEEG" OR "NIRS" OR "near infrared spectro*" OR "NIR spectro*" OR "cell-free DNA" OR "circulating DNA" OR "cfDNA" OR "ccfDNA" OR "DNA methylation patterns" OR "brain-specific DNA" OR "genetic marker*" OR "biomarker*" OR "biological marker*" OR "biologic marker*" OR "serum marker*" OR "laboratory marker*" OR "s100B" OR "s100β" OR "Glial ﬁbrillary acidic protein" OR "GFAP " OR "NSE" OR "Neuron specific enolase" OR "Brain derived neurotrophic factor" OR "BDNF")
